# Gene–methylation interactions: discovering region-wise DNA methylation levels that modify SNP-associated disease risk

**DOI:** 10.1186/s13148-020-00881-x

**Published:** 2020-07-16

**Authors:** Julia Romanowska, Øystein A. Haaland, Astanand Jugessur, Miriam Gjerdevik, Zongli Xu, Jack Taylor, Allen J. Wilcox, Inge Jonassen, Rolv T. Lie, Håkon K. Gjessing

**Affiliations:** 1 Department of Global Public Health and Primary Care, University of Bergen, Bergen, N-5020 Norway; 2 Computational Biology Unit, University of Bergen, Bergen, N-5020 Norway; 3 Centre for Fertility and Health, Norwegian Institute of Public Health, Oslo, N-0213 Norway; 4 Department of Genetics and Bioinformatics, Norwegian Institute of Public Health, Oslo, N-0473 Norway; 5 National Institute of Environmental Health Sciences, Research Triangle Park, 27709 NC USA

**Keywords:** Integrative analysis, Statistical interaction effect, DNA methylation, Genome-wide data, Parent-of-origin, Case-parent triads, Haplin

## Abstract

**Background:**

Current technology allows rapid assessment of DNA sequences and methylation levels at a single-site resolution for hundreds of thousands of sites in the human genome, in thousands of individuals simultaneously. This has led to an increase in epigenome-wide association studies (EWAS) of complex traits, particularly those that are poorly explained by previous genome-wide association studies (GWAS). However, the genome and epigenome are intertwined, e.g., DNA methylation is known to affect gene expression through, for example, genomic imprinting. There is thus a need to go beyond single-omics data analyses and develop interaction models that allow a meaningful combination of information from EWAS and GWAS.

**Results:**

We present two new methods for genetic association analyses that treat offspring DNA methylation levels as environmental exposure. Our approach searches for statistical interactions between SNP alleles and DNA methylation (G ×Me) and between parent-of-origin effects and DNA methylation (PoO ×Me), using case-parent triads or dyads. We use summarized methylation levels over nearby genomic region to ease biological interpretation. The methods were tested on a dataset of parent–offspring dyads, with EWAS data on the offspring. Our results showed that methylation levels around a SNP can significantly alter the estimated relative risk. Moreover, we show how a control dataset can identify false positives.

**Conclusions:**

The new methods, G ×Me and PoO ×Me, integrate DNA methylation in the assessment of genetic relative risks and thus enable a more comprehensive biological interpretation of genome-wide scans. Moreover, our strategy of condensing DNA methylation levels within regions helps overcome specific disadvantages of using sparse chip-based measurements. The methods are implemented in the freely available R package Haplin (https://cran.r-project.org/package=Haplin), enabling fast scans of multi-omics datasets.

## Background

Genome-wide association studies (GWAS) of single-nucleotide polymorphisms (SNPs) have contributed enormously to our understanding of the genetic underpinnings of various complex diseases. However, it has become increasingly clear that the heritability of a disease cannot be fully explained by GWAS alone, prompting researchers to examine rare variants, other types of omics data, and alternative disease mechanisms in an attempt to explain the missing heritability.

The term “epigenome” is widely used to encapsulate all the epigenetic processes involved in regulating gene expression in the entire genome. Epigenome-wide association studies (EWASes) have become a relatively common complementary omics to GWAS as a result of major advances in high-throughput microarray-based technologies for measuring DNA methylation (DNAm) [1, 2]. Among several epigenetic modifications characterized to date, DNAm is by far the most studied epigenetic mark in humans. It is a process by which a methyl group binds to the cytosine (C) at a CpG dinucleotide, resulting in activation or repression of gene expression through mechanisms that are highly region- and context-dependent [3–5]. Furthermore, it has been shown that the state of methylation is controlled by several enzymes [6] and is influenced by environmental exposures [7].

A standard GWAS analysis for a dichotomous phenotype computes relative risks (RRs) between all SNPs and the phenotype. However, it is easy to envision that the effect of a SNP on the phenotype can be modified by DNAm levels in the nearby regions, for instance, when DNAm affects the gene expression. In statistical terms, this corresponds to finding an interaction between the SNP and nearby CpG methylation levels. For instance, the RRs may differ depending on whether DNAm levels are low, medium, or high. Here, we refer to this as a gene–methylation interaction effect (G ×Me).

We are aware of only two studies in the literature that have explored G ×Me effects [8, 9]. The authors of those papers analyzed several SNPs and one CpG in a candidate gene for asthma and detected a statistically significant interaction between a specific SNP–CpG pair. The relative risk of asthma associated with the SNP increased with an increasing level of methylation at the CpG site. Both studies were, however, limited in that they only investigated a few SNPs in one gene and only a single CpG, which were selected a priori because they all showed significant associations with the phenotype. Developing an efficient method that could be applied to the entire genome and epigenome would thus advance the field substantially by enabling an agnostic search for G ×Me interactions.

In addition to G ×Me interactions, it is important to consider parent-of-origin (PoO) effects, which may account for a fraction of the unexplained heritability of a trait. Here, we define a PoO effect as the effect of a particular allele in the child depending on whether the allele is inherited from the mother or the father; see the references and discussion in our previous work [10]. While the main genetic and gene–environment (G ×E) effects can be estimated using a case–control design, assessing a PoO effect requires genetic information from at least one parent, thus a dyad or triad design [11]. An advantage of these family-based designs is that it is possible to estimate G ×E effects even when only the genotypes of the case families are available [12]. The ability to estimate PoO effects opens an entirely unexplored possibility, namely to study how DNAm levels influence PoO effects. Previously, we have developed models for estimating PoO ×E effects in GWAS analyses, where E denotes an external environmental exposure [10, 13, 14]. By letting DNAm levels take the role of the exposure variable, we can thus evaluate PoO ×Me effects, i.e., we can study how the PoO effect changes depending on the nearby DNAm levels.

While G ×Me effects are interpreted analogously to those derived from a cohort or case–control setting, the PoO ×Me interactions offer an intriguing extension. Since genetic imprinting can lead to a PoO effect for a phenotype association, methylation levels at CpGs located near a SNP exhibiting a PoO effect may influence the magnitude of that effect.

In this paper, we treat the level of DNAm as environmental exposure and develop new statistical methods to estimate G ×Me and PoO ×Me interactions from case-parent triads and dyads in a full GWAS-EWAS setting. Our implementation of the methods is applicable to any dichotomous trait where genotypes and methylation status are available on cases (affected children), and the genotypes of at least one parent can be obtained. For each SNP, we investigate DNAm in various genomic regions and not only at a single CpG site because correlated CpGs are known to exert their effects over long stretches of DNA [15–17].

G ×E analyses using case-parent triads are generally robust in the sense that they only require genes and environment to be statistically independent, *conditional on* parental genotypes [18]. This somewhat technical condition would usually be satisfied when E represents an external environmental factor. However, replacing E with Me may be problematic in a few cases, such as when the SNP and a nearby CpG represent an meQTL pair, i.e., where the SNP exerts direct influence on the CpG methylation levels, and thus violates the independence assumption. While this is less of a problem for PoO ×Me interactions, we show how control triads can be used to resolve this issue for G ×Me analyses.

We showcase our methods on orofacial clefts (OFC), which are relatively common congenital malformations with a high heritability and recurrence risk [19]. Several GWASes on OFC have been published, confirming previously reported genes and loci for OFC and identifying new ones for further investigation (for a review, see [20]). Besides the genetic variants, environmental factors have been shown to influence the risk of OFC [21, 22]. A recent study using Mendelian randomization showed that DNAm might mediate genetic liability to clefting [23] and suggested that the causal pathway might proceed in the following direction: environment →*DNAm*→ OFC. In our proof-of-concept analyses described here, we analyzed a well-vetted set of genetic variants known to be strongly associated with OFC risk [24]. This increases the chance of identifying any weaknesses in our theory and minimizes the multiple-testing burden.

## Results

### Implementation of the methods

**Fig. 1:**
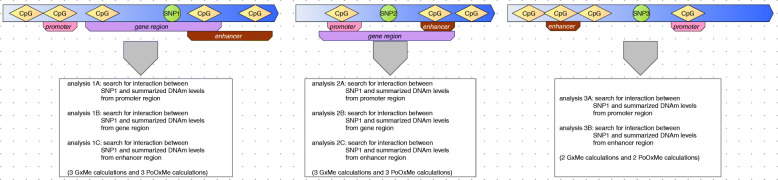
Scheme of the choice of SNP–methylation pairs used in the analyses presented here. For each SNP (green circle), we detect all the CpGs within 50 kb and check which of those are in a promoter, gene, or enhancer region. Note that some of the CpGs can be part of more than one region type, while some have no annotation. Next, we summarize the DNAm values of all CpGs within each region and use this single value to create strata and search for gene–methylation (G ×Me) or parent-of-origin–methylation (PoO ×Me) interaction. For each SNP, we perform six analyses at most

**Fig. 2:**
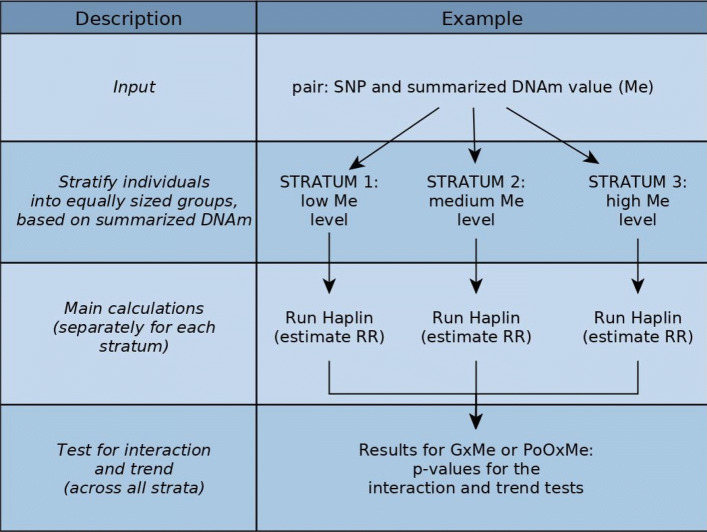
Flowchart of the method for integrating DNA methylation information into genetic association analyses. *RR* relative risk, G ×Me gene–methylation interaction, PoO ×Me parent-of-origin–methylation interaction

As mentioned, the main idea behind our methods is to treat the level of DNAm as environmental exposure in the G ×E or PoO ×E analyses to estimate the change of RR of a disease, depending on DNAm levels. The workflow of the method is presented in Figs. 1 and 2, while the details of the modeling framework, implementation, and analyses are presented in the “Methods” section and in Additional file 2. Below, we give a brief overview of the implementation.

We start by summarizing the DNAm levels from several CpG sites within a specific region located near a given SNP (see Fig. 1). To ease the biological interpretation of the results, we focus on three types of genomic regions: promoter, enhancer, and gene body (for detailed definitions, see the “Genomic regions” section). Because the implementation does not depend on region type, the methods are equally well applicable to, e.g., CpG-rich regions or single CpG sites.

Next, to create discrete categories of the continuous DNAm levels, we average the DNAm levels of the CpGs within each region and divide the individuals into three equally sized strata, based on whether their average DNAm level in a specific region is low, medium, or high. In the “Discussion” section, we show that our method of summarizing the DNAm levels helps to retain important information carried by each CpG site and offsets the disadvantages of using sparse, chip-based measurements of DNAm.

For each DNAm stratum, we estimate the RR of the SNP relative to the trait and then test for trend and interaction between strata (see Fig. 2 and the “Implementation details” section). The interpretation of the G ×Me analysis is that a statistically significant change in RR across strata would indicate an interaction. Similarly, for the PoO ×Me analyses, we perform PoO analyses for each stratum and then check for a significant change across strata.

### Application of the methods

To test our methods, we apply them to genotype data from mother–child dyads and genome-wide DNAm data from the children only. These data are available on controls and cases. Cases are children diagnosed with OFC and divided into following subsets: cleft lip only (CLO), cleft lip with cleft palate (CLP), cleft lip with or without cleft palate (CL/P), or cleft palate only (CPO). We focus the current analyses on the subset of SNPs that showed the strongest associations with OFC risk in a recent study that used the same genetic dataset as here [24] (Table 1). Depending on whether there are any CpGs near a SNP that could be categorized as belonging to one of promoters, enhancers, or genes (Table 2), we conduct the G ×Me and PoO ×Me analyses up to three times for each SNP in the case triads. We also repeat the analyses on control data to see if there are any background correlations between SNPs and DNAm. This would indicate false-positive results due to, e.g., the presence of meQTLs. In the sections below, we present the most significant results. All the *p* values are provided in Tables S1–S5 and S7–S11 in Additional File 1.

**Table 1:** The SNPs selected for the current analyses, along with the names of the nearest genes (if any), and the measures of association (relative risks (RR), 95% confidence intervals (CI), and *p* values; taken from Tables 1–3 in Ref. [24])

**SNP**	**Locus**	**Minor allele**^*a*^	**MAF**^*a*^	**RR**	**95% CI**	***p**** value*	**Cleft subtype**^*b*^
rs12543318	*8q21.3*	c	0.31	1.51	1.31–1.75	4.46e ^−8^	CLO
rs987525	*8q24*	a	0.19	1.85	1.63–2.10	1.47e ^−19^	CLP
rs560426	*ABCA4*	g	0.44	1.24	1.10–1.41	3.91e ^−4^	CLP
rs3758249	*FOXE1*	t	0.36	0.78	0.69–0.88	8.18e ^−5^	CLP
rs642961	*IRF6*	a	0.23	1.60	1.36–1.87	1.41e ^−8^	CLO
rs7078160	*KIAA1598*	a	0.17	1.33	1.15–1.53	1.04e ^−4^	CLP
rs13041247	*MAFB*	c	0.41	0.67	0.59–0.76	2.32e ^−9^	CLP
rs227731	*NOG1*	g	0.47	0.74	0.64–0.85	3.8e ^−5^	CPO
rs742071	*PAX7*	t	0.38	1.52	1.31–1.75	3.74e ^−8^	CLO
rs8001641	*SPRY2*	g	0.54	0.79	0.70–0.90	2.05e ^−4^	CLP
rs7590268	*THADA*	g	0.25	1.27	1.12–1.46	3.96e ^−4^	CLP
rs1873147	*TPM1*	g	0.27	1.31	1.13–1.53	5.82e ^−4^	CLO

^a^The minor allele and its frequency (MAF) for the Norwegian population were taken from Table 1 in the Appendix of Ref. [24]

^b^The cleft subtype (CLO, CPO, or CLP) for which the association was the strongest

**Table 2:** Availability of data for G ×Me and PoO ×Me analyses. We used genotypes and DNA methylation data and classified CpGs into one of gene, promoter, or enhancer classes. In each of those groups, we performed both G ×Me and PoO ×Me analyses, where at least one CpG was localized within 50 kb from the indicated SNP (gray shading; the number indicates how many CpGs were in each category)

	

### G ×Me analyses

**Fig. 3:**
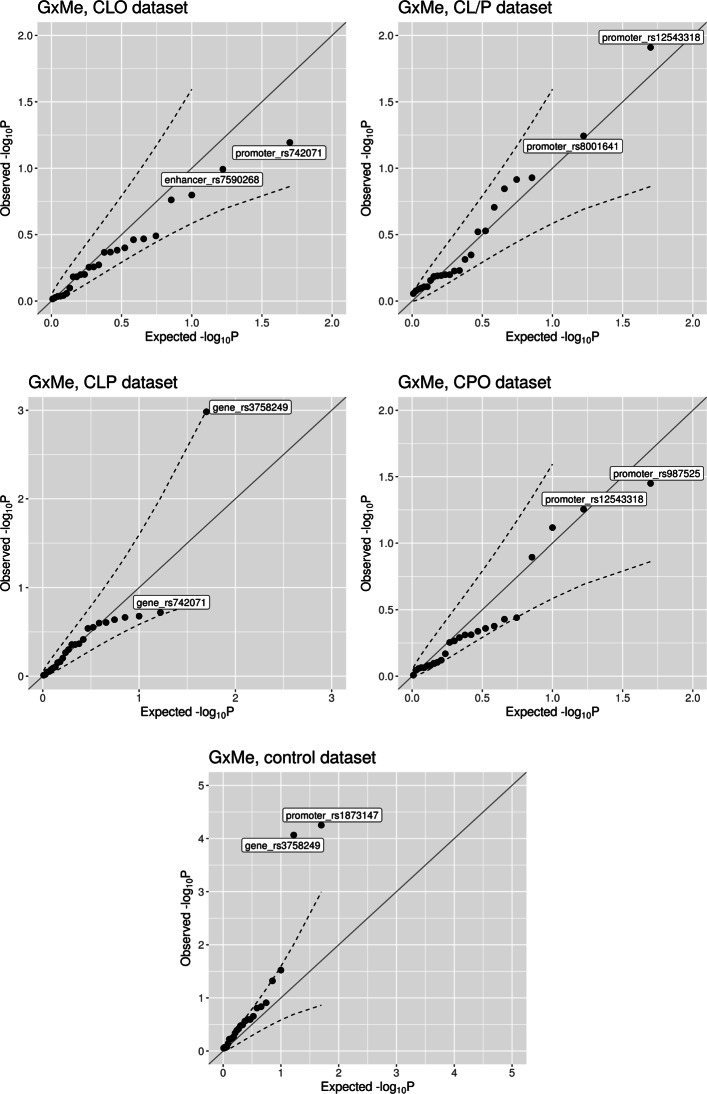
Quantile–quantile plots of the interaction *p* values from the G ×Me analyses. The dashed lines represent the 95% confidence interval

**Fig. 4:**
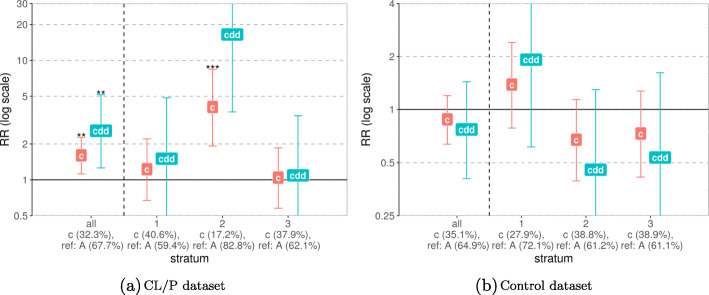
G ×Me effect between rs12543318 at locus *8q21.3* and the methylation level of the CpG from the promoter region nearby. The *x*-axis groups the results into the unstratified dataset “all” and the results for each stratum: “1” denoting low methylation level, “2”—medium, and “3”—high methylation level. The minor and reference alleles are given for each group, along with their frequencies. The *y*-axis shows the relative risk on a log scale, with “c” denoting the child effect when only one minor allele is inherited (single dose) and “cdd” denoting the child effect when two such alleles are inherited (double dose)

The results of the G ×Me analyses are easiest to evaluate when the Wald interaction test *p* values are plotted as quantile–quantile (Q-Q) plots (Fig. 3). The Q-Q plots for CLO and CPO showed no significant interactions. The results for CL/P pointed to a possible interaction between rs12543318 at 8q21.3 and the methylation state of the promoter-flanking region nearby (one CpG, regulatory stable ID: ENSR00001432551 for GRCh37 and ENSR00000861245 for GRCh38). The *p* value of 0.012 indicates a change in the relative risk depending on the methylation status in that region. Figure 4a also shows that the risk of CL/P changes markedly among individuals carrying at least one C-allele at rs12543318 and whose methylation status at the CpG in question was in the middle stratum (i.e., between 0.971 and 0.975). This group had a higher risk of CL/P when the methylation level was taken into account (i.e., in the stratum “2” in Fig. 4a) versus when methylation level was not considered (no stratification, results for “all” in Fig. 4a). Thus, it is plausible that the interaction is driven by the change in RR in the middle stratum. As illustrated in Fig. 4b, the result of the stratified analysis of the control dataset did not show any trend and was not significant.

**Fig. 5:**
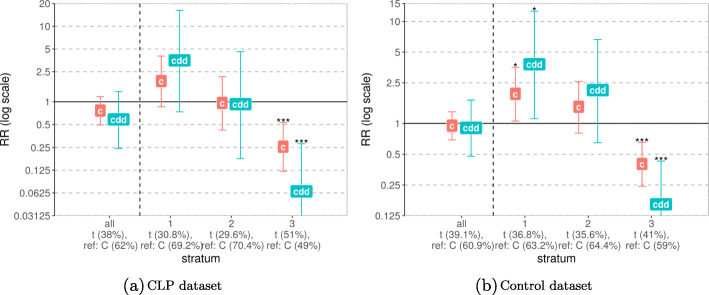
G ×Me effect between rs3758249 in *FOXE1* and the methylation level at the CpGs from the gene region. The *x*-axis groups the results into the unstratified dataset “all” and the results for each stratum: “1” denoting low methylation level, “2” — medium, and “3” — high methylation level. The minor and reference alleles are given for each group, along with their frequencies. The *y*-axis shows the relative risk on a log scale, with “c” denoting the child effect when only one minor allele is inherited (single dose) and “cdd” denoting the child effect when two such alleles are inherited (double dose)

The CLP analysis resulted in one significant interaction between rs3758249 in *FOXE1* and the methylation status of the gene region (Fig. 3, a *p* value of 0.001). Intriguingly, we found the same significant interaction in the control dataset, and the pattern was similar in the analyses of cases and controls (Fig. 5). Therefore, this interaction is most likely a false positive. The results of G ×Me analysis on the control dataset produced another significant interaction, between rs1873147 in *TPM1* and the methylation status of a promoter region nearby (see Fig. S9 in Additional File 1). This was not replicated in any of the case datasets, which could be in part due to sample-size issues (the control dataset is approximately 3 times larger than the case datasets).

**Fig. 6:**
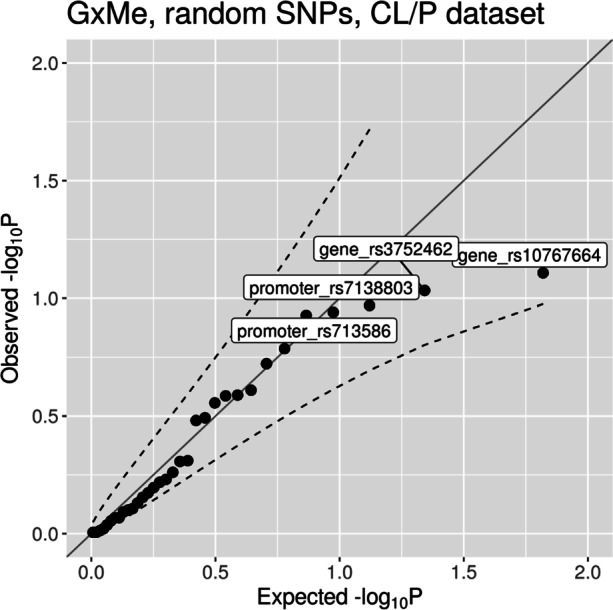
Quantile–quantile plot of the G ×Me results from the analysis of 20 randomly selected SNPs in the CL/P dataset

**Random-SNP analysis** We checked whether the assumptions of the G ×E modeling framework are met when using DNAm data as exposure by randomly picking 20 SNPs from the CL/P data (Table S6 in Additional File 1). We then applied the G ×Me procedure on this dataset to see if there were any false-positive results. As expected, these analyses did not produce any significant *p* values (Fig. 6).

### PoO ×Me analyses

**Fig. 7:**
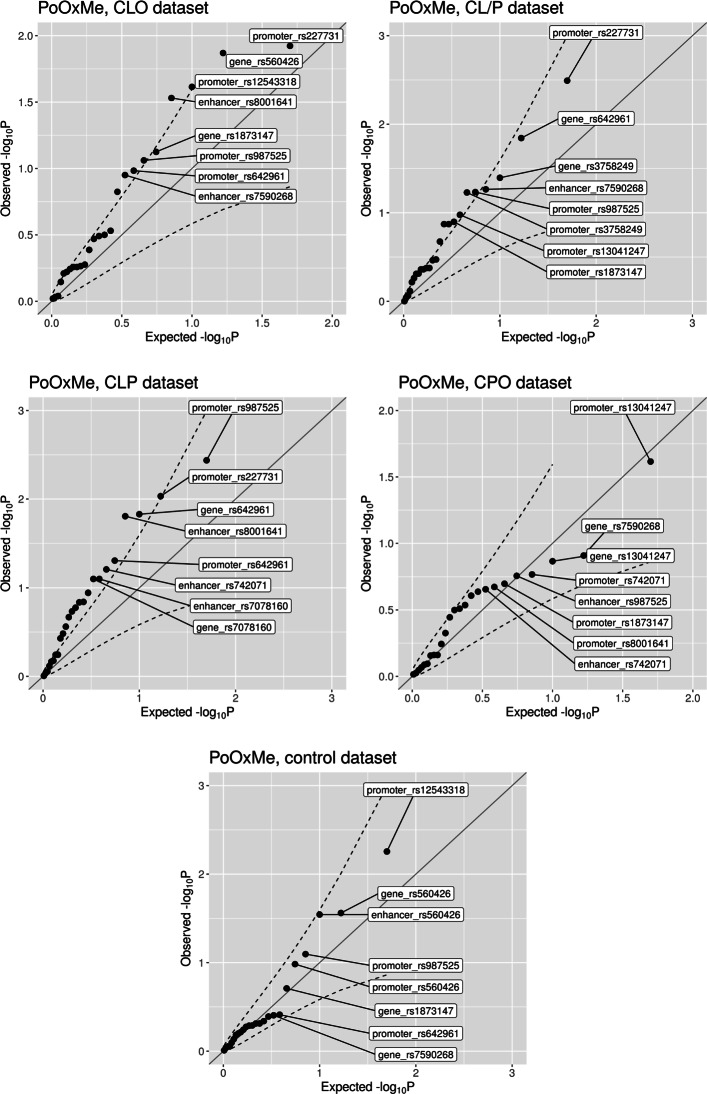
Quantile–quantile plots of the interaction *p* values from the PoO ×Me analyses. The dashed lines represent the 95% confidence interval

**Fig. 8:**
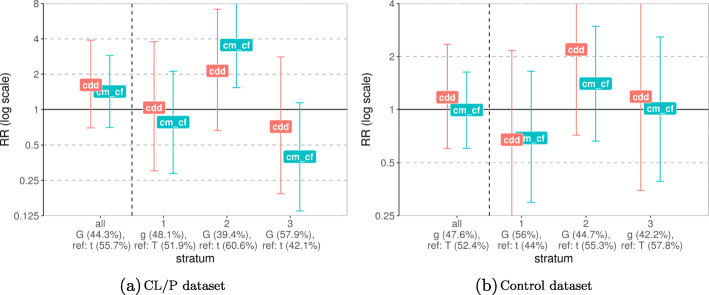
PoO ×Me interaction between a parent-of-origin effect of rs227731 in *NOG1* and the methylation level of the CpGs within a promoter region nearby. The *x*-axis groups the results into the unstratified dataset “all” and the results for each stratum: “1” denoting low methylation level, “2” — medium, and “3” — high methylation level. The minor and reference alleles are given for each group, along with their frequencies. The *y*-axis shows the relative risk on a log scale, with “cm_cf” denoting the parent-of-origin effect when only one minor allele is inherited (single dose) and “cdd” denoting the parent-of-origin effect when two such alleles are inherited (double dose)

There were a few borderline significant PoO ×Me effects in the CLO, CLP, and CL/P datasets (Fig. 7). The most interesting result was the interaction between the PoO effect of the allele at rs227731, located near *NOG1*, and the methylation status of the promoter-flanking region nearby (ensembl regulatory ID ENSR00001131154 for version GRCh37 and ENSR00000559290 for version GRCh38). It is one of the most significant results among the CL/P, CLP, and CLO datasets (Fig. 7). The results displayed in Fig. 8 showed that the risk of CL/P was altered among individuals who had inherited at least one G-allele at rs227731 from the mother and in whom the methylation level in the promoter-flanking region was in the stratum “2” (i.e., average *β* values between 0.51 and 0.52). This group had a higher risk of CL/P compared to the results of the analysis without stratification (the “all” group). Similar patterns were observed for CLO and CLP (Figs. S11a and S12b in Additional File 1).

**Fig. 9:**
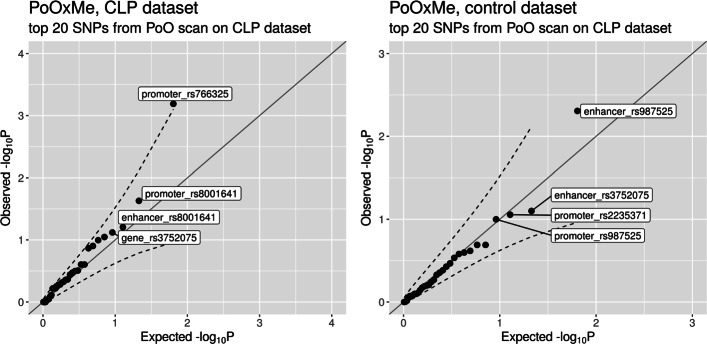
Quantile–quantile plot of the *p* values from the PoO ×Me analysis using the top 20 SNPs with the lowest *p* values from the GWAS scan for PoO effects of the CLP dataset. The dashed lines represent the 95% confidence interval

**PoO scan** We also conducted a scan over all the SNPs in the CLP dataset to search for any significant PoO effects. The top 20 hits from the scan had PoO *p* values in the range 2.1·10^−3^ to 0.27 (see Table S12 in Additional File 1). We then performed PoO ×Me analyses on these top 20 hits in both the CLP and the control datasets (Fig. 9). Only one SNP had a more significant *p* value than would be expected by chance. Namely, the *p* value for the interaction between the PoO effect of rs766325 and the methylation level in the promoter region nearby was above the 95% point-wise confidence interval. The RR increased with increasing methylation level of the promoter (ensembl regulatory stable ID ENSR00000923082) (see Fig. S13 in Additional File 1).

## Discussion

We present here two methods that incorporate DNAm data into genetic association analyses, which we call G ×Me and PoO ×Me. Although a simpler implementation of the method for G ×Me has previously been reported [8], our setup is substantially more comprehensive in scope. We also designed a novel PoO ×Me analysis that requires fewer assumptions than G ×Me. We applied the two methods on genotype and DNAm data from children with OFC and their mothers, as well as from control children and their mothers. Both methods are designed and implemented to facilitate a full genome-wide screening, but for illustration, we conducted a more targeted analysis where we focused on a set of SNPs that had previously shown significant associations with OFC risk. Thus, any change in the relative risk estimate due to the DNAm level should be easily detectable. Note that when judging the significance of the results after running our methods in a full genome scan mode, one can apply any standard multiple-testing method, typically based on controlling the false-discovery rate. This is because both methods return one *p* value per SNP; we do not consider SNP ×SNP interactions, which would produce a large number of correlated test results.

There are many ways of combining genetic and methylation data to predict disease prevalence or risk. For example, Shah et al. [25] tested several linear models to investigate the extent to which disease prevalence was due to genetic or methylation components alone. Further, White et al. [26] performed a multi-step analysis of several omics data to identify genetic associations with neurological disorders. In another work, linear regression models were tested to identify associations between DNA methylation levels at specific CpG sites and child’s birth weight [27]. These results were subsequently used to test for association between the top CpGs and genotypes. A few studies searched for an association between a specific genotype and DNAm at a chosen CpG from a neighboring region [28–30]. Another study adopted a more integrative approach by utilizing all the available genetic and methylation data to search for differentially methylated CpG sites and meQTLs associated with breast cancer [31].

In contrast to the research mentioned above, our methods focus on dyad- or triad-based data, which are robust study designs that are particularly well-suited for early-onset disorders where biospecimens can be collected from both parents and their offspring. Moreover, we use CpG regions, and not only single CpG sites, to perform region-based CpG analyses to capture biologically relevant effects of DNAm on genotype. We implemented our new methods in the well-established R package Haplin. Our entire code is available to other researchers interested in testing and adapting the interaction models to their analyses (see the “Availability of data and materials” section below).

### Summarizing the methylation patterns in regions facilitates the biological interpretation of results

We assessed the DNAm level of CpG regions instead of single CpGs and used the most straightforward method to average the *β* values from all the CpG sites within a region. This single value from each region was then used to divide the study participants into equally sized groups according to the methylation level of each region. Although each CpG can have its unique methylation status, methylation levels of nearby CpGs have been shown to be correlated [15, 32]. Moreover, averaging the methylation level over a region has previously been successfully used to search for differentially methylated regions (DMRs) [33], when imputing missing values [34], or when defining a methylation score for a region [35]. If a given CpG displays a much wider range of methylation levels than other CpGs within the same region, this CpG will have the largest influence on the average methylation value.

However, the strategy of summarizing the methylation pattern in a region has one important caveat. For instance, let us assume that there are only two CpGs in a given region, and that, for two individuals, the *β* values at these CpG sites are 0.75 and 0.25, and 0.25 and 0.75, respectively. Then these two individuals would be placed in the same methylation category because the sum is 1 in both instances. We observed such a phenomenon with the most significant result of the PoO ×Me analyses; namely, the interaction between rs227731 and DNAm levels of the promoter region nearby. There were only two CpGs in this region, and both had narrow and opposite *β* value ranges (see Fig. S3). To check whether this was problematic for our methods, we plotted the *β* values for each individual at each of the CpG sites separately (Fig. S14 in Additional File 1). The plots showed that there was still differential grouping of the individuals into strata, with both of the CpGs exhibiting a wide range of values across all the strata. We then ran the PoO ×Me analyses only for rs227731 and took only the *β* values of one of the promoter-CpGs, i.e., ch.17.52148184R. Table S13 and Fig. S15 show that the results did not change appreciably when we excluded one CpG. Notably, the influence of the methylation values on RR was the same (compared with Figs. 8, S11a, and S12b), whereas the *p* values were slightly higher.

Thus, averaging the methylation level of CpGs appears to be effective at summarizing the methylation level in the context of the analyses presented here. In future work, we will investigate the impact of different parameter choices and assumptions on our methods and explore how the results might be influenced by the use of a different methylation-summarizing method. Importantly, our implementation of the new methods makes them easily applicable to any other methylation-summarizing method, also concerning other genomic regions.

We chose 50 kb as the maximum distance from a given SNP to define the “nearby CpG sites” and to incorporate the promoter and gene regions in the search. Enhancers, in particular, are known to exert their effects across long distances, but the majority of enhancer–gene pairs are still located within 50 kb [36, 37]. In future developments of the methods, we will perform a more exhaustive sensitivity analysis of those parameters.

Our choice of CpGs was guided by the desire to explain the results in a biologically meaningful context. DNA methylation controls gene expression by allowing or preventing specific transcription factors to bind to promoters, enhancers, or gene bodies depending on the biological context [3, 4, 32, 38], which is why we specifically selected these three regions for our analyses here. Anastasiadi et al. [17] investigated changes in gene expression that are associated with changes in the range of DNAm within promoters, gene bodies, and gene body sub-regions. Their results indicated that, when the association is significant, one can use the mean or median value of methylation instead of the entire set of DNAm values. As a gene body may span a large region, it may potentially house so-called cryptic promoters (see, e.g., Ref.[6]), which can regulate the expression of other genes when their methylation status changes. Therefore, taking into account all CpGs within a gene region might lead to increased statistical power.

### Summarizing the methylation patterns in regions may overcome the disadvantages of using data from a standard chip

Because our analyses are based on DNAm data from a microarray chip, we do not have data on all CpG sites within each of the regions considered. However, reassuringly, recent extensive genome-wide analyses of DNAm data suggest that chip-based methods may provide almost as much information as sequencing techniques [39]. Another potential problem when using chip-based DNAm data is the so-called gaps. As shown by Andrews et al. [40], the measured signals can sometimes be attributed not to the methylation itself, but, instead, to a mismatch occurring when a SNP is located within the DNA sequence of the probe. In those cases, plotting the *β* values of one CpG probe for all individuals would produce a multimodal distribution, typically with gaps in the plot. Note that while the standard pre-processing procedures include removing the probes with a SNP within the sequence, each specific dataset might have its own specific SNPs. We checked for such problems in our datasets, as described in Additional File 1, Section S2.2. The gaphunter algorithm identified two probes as being possibly problematic. However, we did not find any correlation between the alleles and methylation levels. The impact of this problem is likely to be negligible on our methods because we took into account not only single CpG sites but summarized the methylation level within a region. Therefore, we chose to retain the data from these probes.

### Control data are helpful in identifying false positives.

Using log-linear models to estimate G ×E and even PoO ×E effects from dyad and triad studies has been thoroughly tested. Our implementation has been shown to control for the type I error rate satisfactorily even when moderate sample sizes are used [10]. As long as the environmental exposure E is exogenous, it is reasonable to assume that the child’s genes and the environment are independent of each other, conditional on parental genotypes. However, as described in the “Methods” section (“Statistical methods” section and Additional File 2), it would still be prudent to ask whether the corresponding conditions (5 and S.7) are satisfied when treating DNAm as exposure. As a simple and general test of the validity of our approach, we ran the full CpG selection strategy and G ×Me analyses on 20 randomly chosen SNPs. This resulted in no false positives, indicating that the CpG selection procedure did not violate the assumption of conditional independence.

As an additional test for identifying false positives among the results for the SNPs in Table 1, we repeated these analyses on the same SNPs in the control dataset. Intriguingly, the results of G ×Me in the control dataset showed a significant interaction between rs3758249 in *FOXE1* and DNAm level in the promoter region nearby, which was also found to be significant in the analysis of the CLP dataset. We also repeated this search for interaction within the control dataset after having removed one CpG that was found in the mQTL database [41], i.e., cg13791254 (results not shown). The resulting *p* value of the Wald test for interaction between rs3758249 in *FOXE1* and the remaining CpGs in the promoter region was 6.4·10^−5^, which is only a modest change from the original value 8.6·10^−5^.

This highlights the importance of including a control dataset to sift through the initial results and to assess whether the detected G ×Me interactions are genuine. The reason for observing this interaction might be that it is not specific for OFC, given that the methylation data were generated using DNA samples from cord blood, and not from tissues that are more relevant for clefts (e.g., buccal epithelial cells or other craniofacial tissue). Nevertheless, the correlation between DNAm levels in blood and lip/palate tissues has been reported to be high [42].

### The G ×Me and PoO ×Me methods are highly versatile

Since the main idea behind the new methods is to search for statistical interaction, the methods can be applied to a wide range of etiologic scenarios. Our implementation specifies the input as a pair consisting of a SNP and a methylation value. However, it is up to the user how these pairs are created, i.e., whether the methylation level is a value from one CpG alone or a summarized value from many contiguous CpGs, whether the CpG is located near or far away from the SNP, as long as the choice makes biological sense. Moreover, the methods are, in principle, applicable to any complex disease or binary trait.

However, several points need to be addressed. These methods generally require samples from many individuals, since they are used to calculate the interaction effect, not the main effect (see, e.g., Fig. 2 in our previous work [10]). Thus, if the disease is highly polygenic, substantially more samples would be required to achieve reasonable confidence intervals for the interaction estimates. Our implementation provides more power per sample due to the calculation of the trend test and because we use only one measure of methylation level within a region instead of analyzing each CpG separately. Moreover, while the Haplin implementation is valid for dichotomous phenotypes, it should be possible to perform the search for interaction effects with software for continuous traits.

Furthermore, in our interaction analyses, we divided the dataset into three strata. This is perhaps the most reasonable minimum number of strata required, where one can apply a trend test and visually assess the changes in RR across strata. To check how sensitive the results were to the number of methylation strata, we also used two and four strata (results not shown). Generally, the four-strata analyses collapsed due to the low number of observations in each stratum and the two-strata analysis was too crude to detect trends. With larger sample sizes, a finer division than three strata may be used.

### The interpretation of the significant gene–methylation interaction is not straightforward

The results of our G ×Me analyses point to an interaction in the CL/P dataset between rs12543318 at 8q21.3 and the methylation state at CpG cg03309455 in the promoter-flanking region nearby (regulatory stable ID: ENSR00001432551 for GRCh37 and ENSR00000861245 for GRCh38). However, it is not easy to interpret the biological relevance of this interaction. According to the JASPAR database [43], two transcription factors (TFs) are predicted to bind to the sequence containing CpG cg03309455: ETS-related gene (ERG; uniprot ID: P11308) and Neurogenic differentiation factor 2 (NEUROD2; uniprot ID: Q15784). While ERG is a general factor expressed in 197 tissue types, according to a search in the bgee database (https://bgee.org/?page=gene&gene_id=ENSG00000157554), NEUROD2 is specifically involved in neuronal determination. rs12543318 itself is located in a non-coding region, with no genomic annotation.

**Fig. 10:**
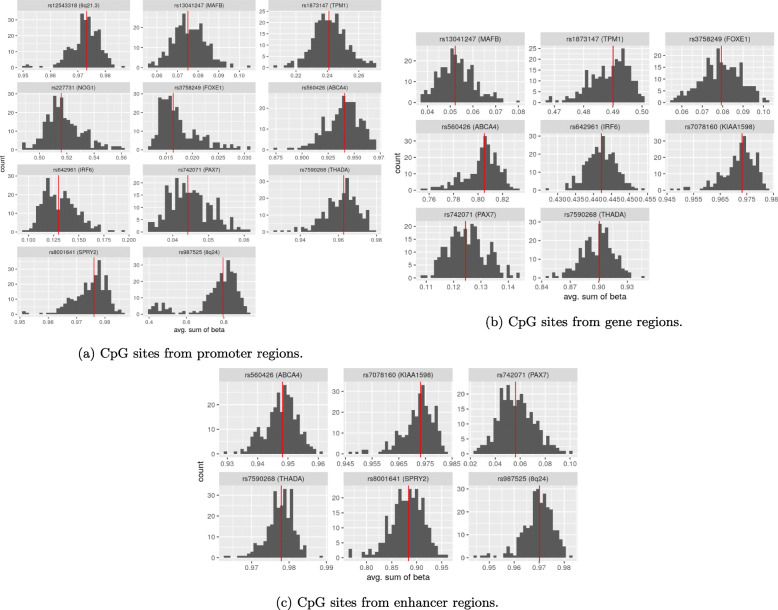
Histograms of the averaged *β* values in the CL/P dataset; the red line indicates the median value

As seen in Fig. 4, the above interaction was found in the CL/P dataset, and there is no matching result in the control dataset. This raises the probability that the interaction is a true positive. However, it should be noted that across the individuals in our dataset, the range of *β* values for this CpG is narrow and higher than 0.95 (Fig. 10a). Hence, this small absolute difference in methylation value across the three methylation strata in the interaction analysis renders a consistent biological interpretation less likely.

### Parent-of-origin effect interaction with DNA methylation was significant near rs227731

There was one significant PoO ×Me interaction in three of the five tested datasets, namely, the PoO effect of rs227731 interacting with the methylation values of the promoter region nearby. There were two CpGs in this region in our data: ch.17.52148184R and cg24806663. According to the newest data in the JASPAR database, there are 19 TFs that most probably bind to the cytosine that is methylated in ch.17.52148184R (Table S14 in Additional File 1). We checked the gene ontology (GO) annotations of these proteins in the QuickGO browser (https://www.ebi.ac.uk/QuickGO/). Of these 19 TFs, 11 were annotated with “multicellular organism development” (GO ID GO:0007275), while six TFs were annotated with “embryonic skeletal system morphogenesis” (GO:0048704). Of note, one of the TFs is Homeobox protein BarH-like 1 (BARX1, UniprotID: Q9HBU1), which is involved in craniofacial development and odontogenesis [44]. The JASPAR database pointed to only four TFs that most likely bind to the cytosine in cg24806663 (Table S15 in Additional File 1), three of which are involved in transcriptional repression.

At the same time, Fig. 8a shows that this interaction is due to a noticeable change in the parent-of-origin-specific relative risk among the individuals within the middle stratum of the DNAm levels. However, since we have *β* values that come from chip measurements, it might be that they do not adequately capture the PoO-specific methylation distribution among the cell types and DNA strands.

### The new PoO ×Me method is robust and can point to possible imprinting issues

The PoO ×Me method presented here has a more relaxed requirement on the independence between methylation levels and genotype, as outlined below (the “Statistical methods” subsection of the “Methods” section). Furthermore, it makes full use of the dyad and triad designs. As we have shown previously [45], there are some advantages of using the dyad design instead of the full triads, as dyads sometimes provide higher statistical power relative to the number of genotyped individuals. However, since the measured *β* values represent an average of DNAm levels from several cell types and, importantly, an average from the two DNA strands, some issues may remain. One is whether we can correctly capture the interaction between PoO and the averaged methylation level; that is, how sensitive is the PoO effect in relation to methylation? A recent study detailing DNA methylome dynamics in early embryonic development [46] showed that the father’s DNAm pattern may be very different from the mother’s during this early development. Only a few genes were found whose expression patterns matched the DNAm differences. It is thus likely that DNAm alone cannot induce significant changes in expression, but is rather associated with variation in gene expression [47].

Our approach to studying PoO ×Me effects is closely related to the contribution of methylation to imprinting. An imprinted locus can be seen as a locus where methylation levels in the child may depend on the parent of origin of the DNA strand. This may potentially lead to an up- or downregulation of the expression of alleles on that strand in a PoO-specific manner. There are many approaches to identify genes that exhibit imprinting [48]. For example, a recent study analyzed combinations of DNAm and genotypes in the child from mother–child dyads [49]. The authors first used maternal genotypes to establish the parent-of-origin status of SNP alleles in the child’s DNA and then searched for SNPs that are associated with methylation status of nearby CpG sites in a parent-of-origin-specific manner. In our notation, this corresponds to finding loci where

$$P(Me|C_{ij}) \neq P(Me|C_{ji}),$$

that is, where the distribution of methylation values at the relevant CpG depends not only on the SNP alleles themselves but also on which parent they were inherited from. Interestingly, this is closely related to our assumption (5) (see the “Statistical methods” subsection of the “Methods” section), which we use to exclude possible false positives by checking the condition in control families. While the approach of Cuellar Partida et al. [49] is not related to a specific phenotype, our analyses are focused on OFC. We thus look for parent-of-origin-specific correlations between the methylation level (*Me*) and the child’s genotype (*C*) that are present among case-children but not among control-children.

## Conclusions

This study implemented two new strategies to search for interactions between DNAm levels and either the genotype (G ×Me) or parent-of-origin (PoO ×Me) effects. In addition, we demonstrate the use of region-wise methylation levels by focusing on biologically meaningful genomic regions (promoter, gene body, and enhancer). The inclusion of these methods in our R package Haplin facilitates the ease-of-use and adaptation, as the code is open-source and free. Additionally, we performed several sensitivity analyses to test the robustness of our methods. While the triad and dyad designs allow all of the analyses presented here to be performed, we note that independent control triads or dyads are important to check and correct for false-positive results, particularly for the G ×Me model.

## Methods

### Genotypes

Genotypes from child–mother dyads were available for cases (1311 dyads) and controls (2481 dyads). Details regarding data collection and quality checks have been provided in our previous work [24] and are summarized in Additional File 1. In the case dyads, the child was diagnosed with one of the following three subtypes of OFC: cleft lip only (CLO), cleft lip with cleft palate (CLP), or cleft palate only (CPO). In addition to these, we also analyzed another category of cleft—cleft lip with or without cleft palate (CL/P), which is a combination of CLO and CLP. For the current analyses, we use a subset of the original dataset by selecting only those families for whom DNAm data were also available for the child.

### DNAm data

The Illumina HumanMethylation 450K BeadChip (Illumina, Inc., San Diego, CA) was used to assess DNAm levels at 485,577 CpG sites in the children from the dyads mentioned above. Details on how the raw data were processed are available in our recent work [50] and are summarized in Additional File 1. The *β* value corresponding to the methylation level at each CpG site was calculated as *β*=*I*_*M*_/(*I*_*M*_+*I*_*U*_+100), where *I*_*U*_ and *I*_*M*_ are the intensities of the unmethylated and methylated signals, respectively, and the factor 100 is added to ensure no division by zero. After the quality control, 407,513 CpGs and 868 samples (456 controls; 105 CLO; 167 CLP and 140 CPO) remained for analysis. Matching of the children from the methylation dataset to the genotype dataset yielded 103 CLO dyads, 269 CL/P dyads, 140 CPO dyads, 166 CLP dyads, and 456 control dyads.

To inspect the entire methylation data and check for inconsistencies, we (i) performed a statistical summary of methylation levels in the regulatory regions (promoter, enhancer, gene body) and (ii) displayed the distributions of the methylation levels around the chosen SNPs. More extensive details on these analyses are provided in Additional File 1 (see Sections S2.1, S2.2, and S2.3). Overall, the distribution of the methylation levels in our data was consistent with that of other published datasets [51].

### Genomic regions

CpG sites located within a maximum distance of 50 kb from each SNP were selected and categorized into specific regions (promoter, enhancer, gene body) using the newest information from the ensembl database [52]. The R package biomaRt [53] was used to extract information from the ensembl Regulatory Feature database (using GRCh37), which includes the positions of promoters and enhancers in the human genome. Based on this information, we classified a CpG into the category “promoter” when the search returned “promoter” (Sequence Ontology [54] accession number SO:0000167) or “promoter_flanking_region” (SO:0001952). The category “enhancer” corresponded to the “enhancer” description (SO:0000165). Gene regions were derived from the ensembl genome browser (GRCh 37) via biomaRt. Because the positions of the CpGs and SNPs in our raw data were based on the human genome hg19/GRCh37 release, we did not use the newest version of the human genome assembly (hg38/GRCh38) to avoid discrepancies in positional information.

To summarize the DNAm levels per region, we averaged the *β* values from the CpG sites within each regulatory region. Figure 10 presents the distributions of these summarized DNAm levels (Me) per region for the largest dataset (CL/P). Almost all of the Me distributions have a normal-like shape, except for the promoter region near rs987525. The distributions within the other datasets are presented in Figs. S5–S8 in Additional File 1.

### Implementation details

We used our R package Haplin [10, 55] to calculate relative risks with confidence intervals for each SNP and for each cleft category using a log-linear model (haplinStrat function). The significance of interactions was estimated using the Wald test (gxe function). Assuming a dose–response relationship across strata, we also tested for a trend to increase power. This approach has been described elsewhere [56]. The scan for PoO effects was performed using the haplinSlide function, with the “window size” parameter set to 1. The R code used for the current analyses is available on the Bitbucket server (see the “Availability of data and materials” section).

We use the multiplicative scale rather than the additive scale when assessing interactions. This decision is partly dictated by the relative risk being the measure of choice for case-parent triads [55, 57]. Moreover, if significant interactions are detected on a multiplicative scale, they will, typically, also be significant on an additive scale; in that respect, our results are conservative.

We used the R packages ggplot2 [58] and ggrepel [59] and the ensembl browser (http://www.ensembl.org) to create the plots. The schemes (Figs. 1 and 2) were created using the yEd software (https://www.yworks.com/products/yed).

### Statistical methods

The full framework behind the new methods is presented in Additional File 2. Here, we highlight the most salient details.

#### G ×Me interactions

Let *M*, *F*, and *C* denote the genotypes of the mother, father, and child, respectively, within a family triad. Here, *M*, *F*, and *C* will refer to a single SNP, but could also refer to, for instance, haplotypes built from a few SNPs in close linkage disequilibrium. In particular, let *C*_*ij*_=*A*_*i*_*A*_*j*_ denote an ordered child genotype, where allele *A*_*i*_ is inherited from the mother and *A*_*j*_ from the father. *C* denotes the corresponding unordered genotype. Let *D* denote that the child has the disease in question and $\bar {D}$ that the child is healthy. Let *Me* denote methylation levels in the child, which may here be low, medium, or high, as described above. We refer to the families where *Me*=*m* as belonging to stratum *m*. The penetrance function is *P*(*D*|*C*_*ij*_,*Me*), which describes how the probability of disease depends on the child’s genotype as well as the level of methylation. The penetrance may depend on parent of origin since *P*(*D*|*C*_*ij*_,*Me*) may differ from *P*(*D*|*C*_*ji*_,*Me*). Specifically, we assume that

1 $$ P(D|C_{ij},Me=m)=B^{(m)}\cdot RR_{M,i}^{(m)}\cdot RR_{F,j}^{(m)},  $$

where *B*^(*m*)^ is a baseline risk in stratum *m*, $RR_{M,i}^{(m)}$ is the relative risk associated with inheriting allele *A*_*i*_ from the mother in stratum *m*, and similarly $RR_{F,j}^{(m)}$ is the relative risk associated with inheriting allele *A*_*j*_ from the father in stratum *m*. We thus assume a multiplicative dose–response model for the allele dose. To make parameters identifiable, we assume $RR_{M,1}^{(m)}=RR_{F,1}^{(m)}=1$ for all *m*, i.e., we choose *A*_1_ as the reference allele in all strata; $RR_{M,i}^{(m)}$ and $RR_{F,i}^{(m)}$ should be estimated from the model when *i*≠1. More details on models that allow deviations from the multiplicative dose–response can be found elsewhere [55, 60].

To look for G ×Me effects, we first assume that $RR_{M,i}^{(m)}$ and $RR_{F,i}^{(m)}$ are equal for all *i* and *m*, i.e., that the risk does not depend on the parent of origin, and let

$$RR_{i}^{(m)}=RR_{M,i}^{(m)}=RR_{F,i}^{(m)}. $$

A G ×Me effect on the risk of disease would mean that the relative risk $RR_{i}^{(m)}$ changes over strata of *Me*=*m* for one or more *i*’s.

If data have been sampled as case-parent triads and control-parent triads, we only observe the distributions *P*(*M*,*F*,*C*,*Me*|*D*) and $P(M,F,C,Me|\bar {D})$, that is, the joint distributions of triad genotypes and methylation strata among the case-parent triads and control-parent triads separately. The number of triads in each category of genotypes and methylation strata is modeled as a log-linear model, and the model parameters are estimated through maximum likelihood [55, 60]. Since for the case triads,

2 $$ {{}\begin{aligned} &P(M,F,C,Me|D)\\&\quad\,=\,\frac{P(D|M,F,C,Me)P(Me|M,F,C)P(C|M,F)P(M,F)}{P(D)}, \end{aligned}}  $$

there is a direct relationship between the parameters in the log-linear model and the penetrance model. However, the model also includes the population parental genotype distribution *P*(*M*,*F*), the straightforward Mendelian transmission part *P*(*C*|*M*,*F*), and the population disease prevalence *P*(*D*). The latter may not be known, but enters the model as a scaling factor.

The basic case-parent and control-parent triad models are formulated in terms of child and both parents. However, the Haplin implementation allows mother–child and father–child dyads to be fitted within the same framework. This is achieved by using the expectation-maximization (EM) algorithm to impute the missing parent and basing inference on the likelihood that accounts for missing data. Further details on how the log-linear models is implemented can be found elsewhere [55, 56].

The factor *P*(*Me*|*M*,*F*,*C*), i.e., the population distribution of methylation within genetic strata, is crucial in our analyses. When independent control triads are available, we make an assumption—for simplicity—of rare disease. In that case,

3 $$ P(M,F,C,Me|\bar{D})\approx P(Me|M,F,C)P(C|M,F)P(M,F).  $$

Thus, when controls are available, there is no need to make specific assumptions about *P*(*Me*|*M*,*F*,*C*) since it can be estimated from control triads. With only case-parent triads available, however, it is clear that assumptions about *P*(*Me*|*M*,*F*,*C*) may interfere with inference on *P*(*D*|*M*,*F*,*C*,*Me*) when observing *P*(*M*,*F*,*C*,*Me*|*D*) only. In Additional File 2, we present the details on the most important situations where this becomes relevant.

#### PoO ×Me effects

PoO effects have been described in different ways in the literature—here, we measure it as a ratio of relative risks (*RRR*). Specifically, if *RR*_*M*_ and *RR*_*F*_ are the relative risks associated with the maternally and paternally derived alleles, respectively, then the PoO effect within a stratum is *RRR*=*RR*_*M*_/*RR*_*F*_. See Gjerdevik et al. [10] for further details on the PoO definition.

To estimate parent-of-origin effects, we now assume that *RR*_*M*,*i*_ is not necessarily equal to *RR*_*F*,*i*_. In other words, the effect of allele *A*_*i*_ on offspring risk may depend on the parental source of the allele. Let *RRR*_*i*_=*RR*_*M*,*i*_/*RR*_*F*,*i*_ be the ratio of the relative risks associated with allele *A*_*i*_. Thus, *RRR*_*i*_≠1 is indicative of a parent-of-origin effect. Let $RRR_{i}^{(m)}$ be the value of *RRR*_*i*_ estimated in stratum *Me*=*m*, and *C*_*ij*_ as defined above. As above, we assume *P*(*D*|*M*,*F*,*C*_*ij*_,*Me*)=*P*(*D*|*C*_*ij*_,*Me*). To be able to estimate the ratio $RRR_{i}^{(m)}$ within each stratum, we again need to make an assumption

4 $$ P(Me|M,F,C)=P(Me|M,F),  $$

i.e., that the methylation in the child may be associated with (parental) genotypes in the population, but that the child’s genotype does not directly influence methylation, conditional on parental genotypes. However, it will now suffice to assume that

5 $$ P(Me|M,F,C_{ij})=P(Me|M,F,C_{ji})=P(Me|M,F,C),  $$

i.e., the child’s genes may influence methylation levels directly, even within parental mating types, but the mechanisms by which the child’s genotype influences methylation values should not depend on whether specific alleles were inherited from the mother or from the father. Under condition (5),

$$\begin{aligned} P(M,F,C_{ij},Me|D) & =\frac{P(D|C_{ij},Me)P(Me|M,F,C_{ij})P(M,F,C_{ij})}{P(D)}\\ = & \frac{P(D|C_{ij},Me)P(C|M,F,Me)P(M,F|Me)P(Me)}{P(D)}\\&\cdot\frac{P(C_{ij}|M,F)}{P(C|M,F)}. \end{aligned} $$

Note that the last fraction is again determined by standard Mendelian inheritance. In a stratum-specific log-linear model, we can still obtain estimates of $RR_{M,i}^{(m)}$ and $RR_{F,i}^{(m)}$ for each stratum *m*. These estimates may be biased since *P*(*C*|*M*,*F*,*Me*) may depend on both *Me*=*m* and the (unordered) *C*=*A*_*i*_*A*_*j*_. However, the ratio $RRR_{i}^{(m)}=RR_{M,i}^{(m)}/RR_{F,i}^{(m)}$ will be unbiased as long as condition (5) is satisfied.

Again, if control triads are available, under the rare disease assumption, we can use estimates from the control triads to check the symmetry assumption (5), or in a combined (hybrid) analysis, unbiased estimates of $RR_{M,2}^{(m)}$ and $RR_{F,2}^{(m)}$ can be obtained.

## Supplementary information

**Additional file 1** File containing supplementary figures and tables.

**Additional file 2** Details of the statistical methods.

## Data Availability

The genetic data that support the findings of this study were described in detail in Ref. [24] and are available to researchers after the application for access to each of the specific data sources. The DNA methylation data that support the findings of this study were measured as described in Ref. [50] and are available from the corresponding author of that paper on reasonable request. Haplin is implemented as a standard package in the statistical software R [61] and can be installed from the official R package archive, CRAN (https://CRAN.R-project.org/package=Haplin). More details about Haplin can be found on our website, https://people.uib.no/gjessing/genetics/software/haplin. The complete R code to run the analyses described here is available on Bitbucket at https://bitbucket.org/jrom/dna_methyl_manuscript_supplementary/, with detailed documentation and raw outputs from the scripts on https://jrom.bitbucket.io/dna-methyl-manuscript-suppl.

## Abbreviations

CL/P: Cleft lip with or without cleft palate
CLO: Cleft lip only
CLP: Cleft lip with cleft palate
CpG: Cytosine-guanine dinucleotide
CPO: Cleft palate only
DMR: Differentially methylated regions
DNAm: DNA methylation
EM: Expectation-maximization
EWAS: Epigenome-wide association study
G ×Me: Gene–methylation interaction effect
GO: Gene ontology
GWAS: Genome-wide association study
meQTL: Methylation quantitative locus
OFC: Orofacial clefts
PoO: Parent-of-origin effect
PoO ×Me: Parent-of-origin–methylation interaction effect
RR: Relative risk
RRR: Relative risk ratio
SNP: Single-nucleotide polymorphism
TF: Transcription factor
