# Supplementary material for: Gene–methylation interactions: discovering region-wise DNA methylation levels that modify SNP-associated disease risk
Source: Clin Epigenetics. 2020 Jul 16;12:109. doi: 10.1186/s13148-020-00881-x (PMC7367265; doi:10.1186/s13148-020-00881-x)
Supplement: Supplementary file 1 — Additional file 1 File containing supplementary figures and tables. [file 13148_2020_881_MOESM1_ESM.pdf]

Gene-methylation interactions: Discovering region-wise DNA methylation levels that modify SNP-associated disease risk

Julia Romanowska[1,2,4], Øystein A. Haaland[1], Astanand Jugessur[1,3,4], Miriam Gjerdevik[1,3], Zongli Xu[5], Jack Taylor[5], Allen J. Wilcox[5], Inge Jonassen[2], Rolv Terje Lie[1,4], Håkon K. Gjessing[1,4]

[1] *Department of Global Public Health and Primary Care, University of Bergen, Bergen, N-5020, Norway;* [2] *Computational Biology Unit, University of Bergen, Bergen, N-5020, Norway;* [3] *Department of Genetics and Bioinformatics, Norwegian Institute of Public Health, Oslo, N-0473, Norway;* [4] *Centre for Fertility and Health, Norwegian Institute of Public Health, Oslo, N-0213, Norway;* [5] *National Institute of Environmental Health Sciences, Research Triangle Park, NC 27709, USA*

Additional File 1

Contents

S1 Summary of genetic and epigenetic data preprocessing . . . **2**

S2 Methylation distribution . . . **3**
S2.1 Methylation levels in promoter and enhancer regions . . . 3
S2.2 Methylation levels around the chosen SNPs . . . 3
S2.3 Distributions of the β values in the chosen regions. . . . 6

S3 Main results . . . **11**
S3.1 G×Me . . . 11
S3.1.1 CL/P dataset . . . 11
S3.1.2 CLO dataset . . . 11
S3.1.3 CLP dataset . . . 12
S3.1.4 CPO dataset . . . 12
S3.1.5 Control dataset . . . 13
S3.1.6 Randomly chosen SNPs, CL/P dataset . . . 14
S3.2 PoO×Me . . . 15
S3.2.1 CL/P dataset . . . 15
S3.2.2 CLO dataset . . . 16
S3.2.3 CLP dataset . . . 16
S3.2.4 CPO dataset . . . 19
S3.2.5 Control dataset . . . 19
S3.2.6 PoO scan, CLP dataset . . . 20
S3.2.7 Checking the significant PoO×Me interaction promoter—rs227731 . . . 21
S3.2.8 Transcription factors binding to CpGs within promoter region near rs227731 . . . 23

S1 Summary of genetic and epigenetic data preprocessing

The genetic data were taken from the Norway Facial Clefts Study (NCL), as part of the data collected for the study in Ref.[1]*. All the details of the data collection, quality control, and preprocessing are available in the Appendix of the cited work. Here, we provide the most salient details. Genotyping was done on a Dynamic Array using competitive allele-specific PCR KASPar chemistry (KBioscience Ltd., Hoddesdon, UK) on a Fluidigm (Fluidigm Corp., South San Francisco, CA, USA) nanofluidic platform. Genotype calling was done using the Fluidigm SNP genotyping software, version 4.1.2, with the default settings (including a non-template control normalization method, and a K-means clustering method). The quality check included setting the confidence threshold to 65% for the genotype calling algorithm and then visually checking all the genotyping plots. The resulting SNPs had high call rates (mostly 98%), all passed the Hardy-Weinberg disequilibrium test at* $P < 10^{-4}$*, and their minor allele frequencies were* $> 2\%$*.*

The DNA methylation was measured on a subsamble from the NCL case- and control-dyads, as detailed in Ref.[2]*. Here, we provide a summary of the measurement and quality control. Autopure LS (Gentra, Minneapolis, MN) was used to extract genomic DNA from whole blood samples, followed by quantification using Quant-iT*^{TM} *PicoGreen dsDNA reagents (Invitrogen). Next, for each sample, 1* μ*g of DNA was bisulfite converted using the EZ-DNA Methylation kit (Zymo Re- search, Irvine, CA) and placed randomly on ten 96-well plates (Illumina HumanMethylation450 BeadChip; San Diego, CA). The DNA methylation level was then estimated at 485,577 CpG sites and the R package illuminaio (ver. 0.18)*[3] *was used to extract the raw probe intensity values. These raw values were preprocessed using ENmix R package*[4]*. The following criteria were used to identify low-quality samples: (1) average intensity value across internal control probes less than 5500, (2) more than 5% of CpG probes having low-quality data (Illumina detection P value* $> 10^{-6}$*, read from less than 3 beads, or outlier value for the probe in the dataset), and (3) clear outliers based on visual inspection of a density plot of total intensity. This led to exclusion of 29 samples. Moreover, one sample was excluded due to sex ambiguity. Next, the low quality CpG probes were defined as follows: (1) more than 5% low-quality data; (2) common SNP within the probe's sequence (minor allele frequency* ≥ 0.05 *in Europeans based on 1000 Genomes Project data), or probes mapping to multiple genomic locations, or CpGs on X or Y chromosomes; (3) CpGs with multiple mode distributions identified with ENmix. The final number of good quality probes was 407,513 CpGs.*

S2 Methylation distribution

S2.1 Methylation levels in promoter and enhancer regions

We summarized the methylation levels in the promoter and enhancer regions. The promoter regions in our dataset were mostly unmethylated and differed significantly from the highly-methylated non-promoter regions (Figure S1a). In contrast, while the enhancer regions were almost all highly methylated, the methylation level of the non-enhancer CpGs spanned the entire range (Figure S1b).

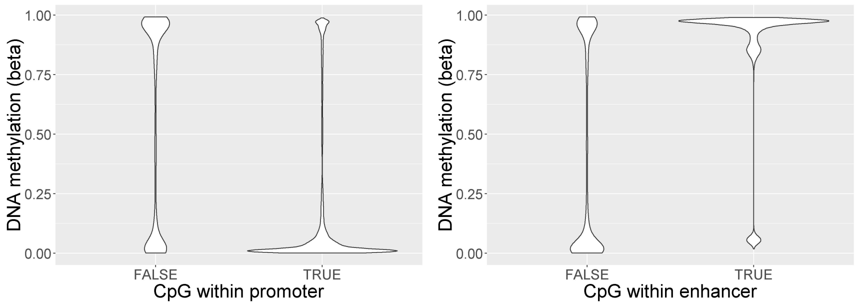

(a) Promoter regions **(b)** Enhancer regions

Figure S1: Comparison of DNA methylation levels at specific regions, shown here as violin plots of β-values per CpG, across all individuals.

As described in the literature[5,6], the promoter regions are usually unmethylated in genes that are being transcribed. This differs from enhancer regions, where the methylation may be correlated with increased gene transcription. In our dataset, the general methylation patterns of the mentioned regions are consistent with these patterns.

S2.2 Methylation levels around the chosen SNPs

We focused our analyses on several SNPs and chose CpGs in their vicinity (Figure S2). First, we analyzed the overall methylation patterns in these regions (Figure S3). Because our data was measured on a chip, it includes only a selected set of CpG sites. Thus, each of the mentioned categories have a distinct number of CpG sites by design, with the CpG sites within gene bodies mostly outnumbering those in the other categories (not including the CpGs that do not belong to any of those categories; Table 2 in the main text).

Generally, the range of methylation level for each CpG site was quite narrow, but there were several interesting exceptions, e.g., cg04350215 near rs560426 in *ABCA4* or cg0164961 near rs7590268 in *THADA* (Figure S3). Moreover, there are several CpGs for which the distribution has "gaps". These need to be investigated, as described in several studies[7,8]. We applied the `gaphunter` function as implemented in `minfi` R package[9] and found two CpGs that were non-unimodal: cg01921066 and cg04350215, both near SNP rs560426 in the *ABCA4* gene body (see Fig. S3). Sometimes this may happen as a result of a SNP being located within the probe[7]. However, we found no SNPs within the sequence of these probes, neither for the population in general (in the ensembl browser), nor within our datasets. We also checked whether the allele of SNP rs560426 influenced the methylation value of these CpGs but did not find such evidence (Figure S4).

If one focuses on all the CpG sites with an annotated regulatory feature, one can see that their methylation levels, overall, span a broad range of the β values, except for the data near rs8001641 in *SPRY2*, rs7078160 in *KIAA1598*, and rs12543318 in 8q21.3. This suggests that considering only one CpG may lead to false conclusions.

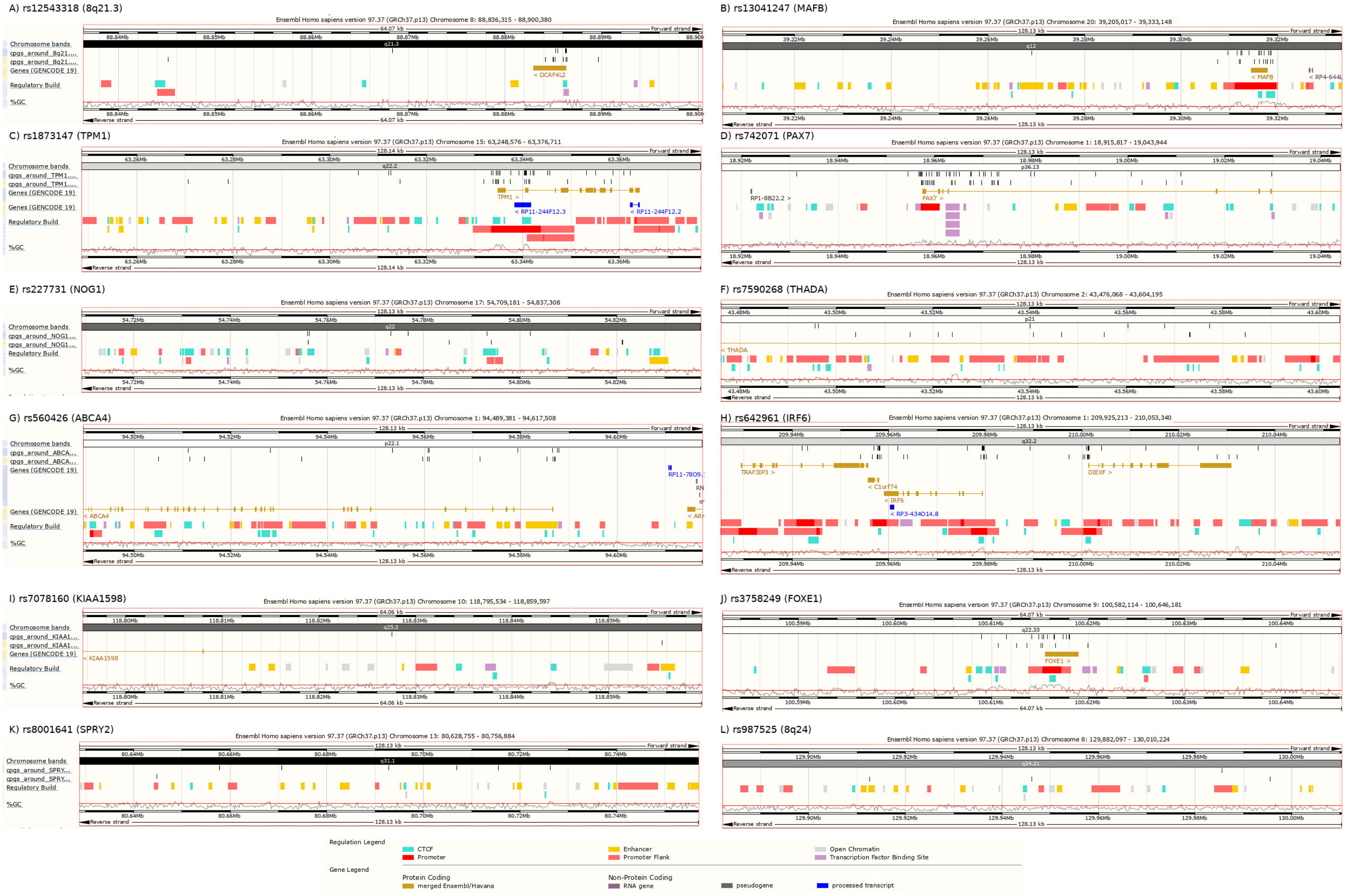

Figure S2: Genomic neighborhood of each of the chosen SNPs. The figures were generated in the ensembl genome browser and display several important tracks:
1. *Chromosome bands*;
2. *cpgs_around_...* — a custom track showing the positions of CpGs and the SNP as black lines, where the SNP is always in the very middle;
3. *Genes (GENCODE 19)* — the genes (if any) shown as colored rectangles (exons) and lines (introns);
4. *Regulatory build* — regulatory regions (if any) shown as colored rectangles;
5. *%CG* — running average of % of CG dinucleotide.
The colors are defined in the legend at the bottom of the figure.

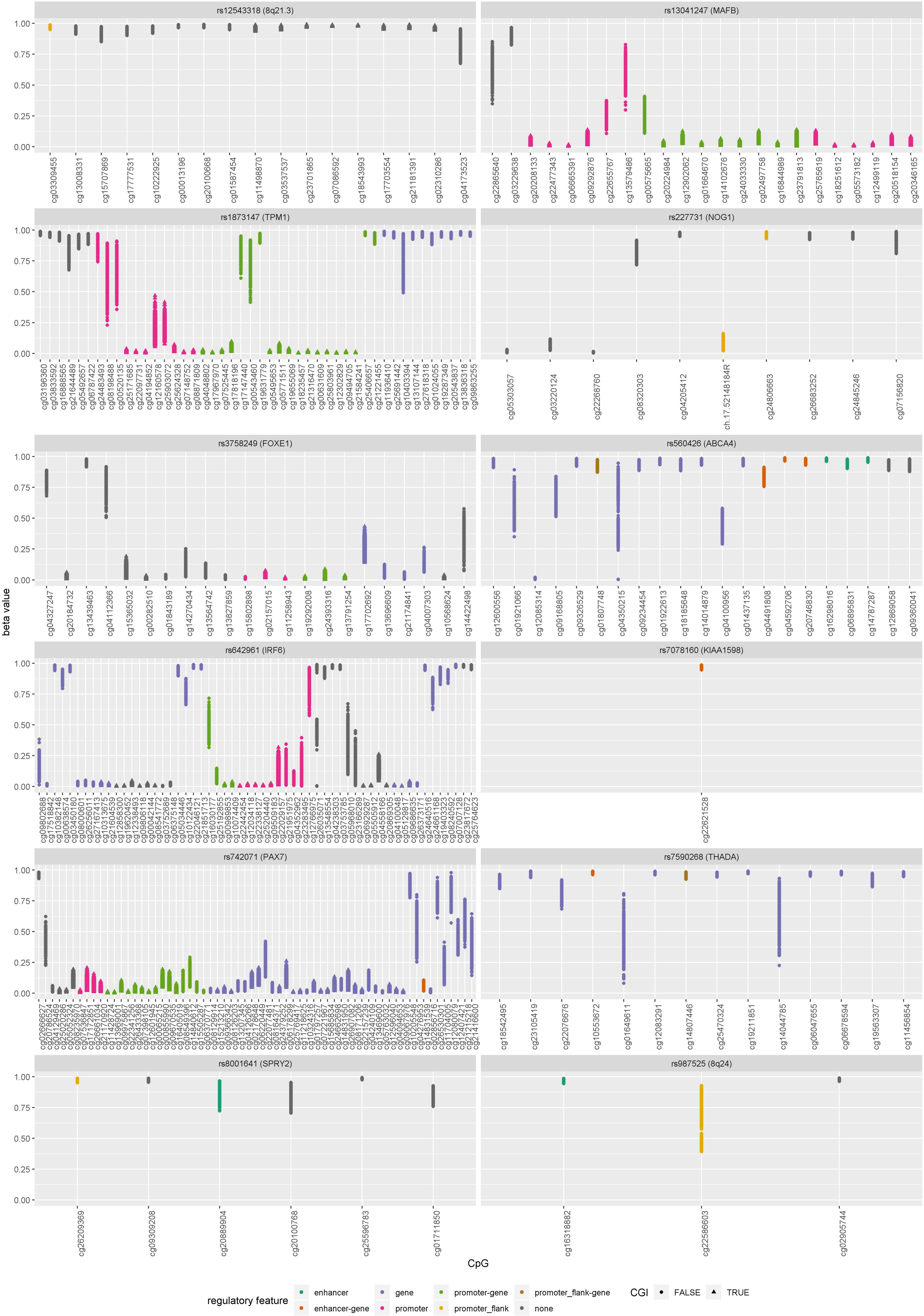
rs12543318 (8q21.3)
rs13041247 (MAFB)
rs1873147 (TPM1)
rs227731 (NOG1)
rs3758249 (FOXE1)
rs560426 (ABCA4)
rs642961 (IRF6)
rs7078160 (KIAA1598)
rs742071 (PAX7)
rs7590268 (THADA)
rs8001641 (SPRY2)
rs987525 (8q24)
beta value
1.00
0.75
0.50
0.25
0.00
CpG
regulatory feature
enhancer
enhancer-gene
gene
promoter
promoter-gene
promoter_flank
promoter_flank-gene
none
CGI
FALSE
TRUE

Figure S3: *(prev. page)* Methylation β-values for CpGs within the 50 kb regions around the SNPs significantly associated with facial clefts; the CpG names are ordered by their positions. The points are colored according to their regulatory role and their shapes show whether the CpG is within a CGI or not. If a CpG site was found to be both within a gene region and in a regulatory region, it was flagged as “promoter–gene”, “promoter_flank-gene” or “enhancer-gene”.

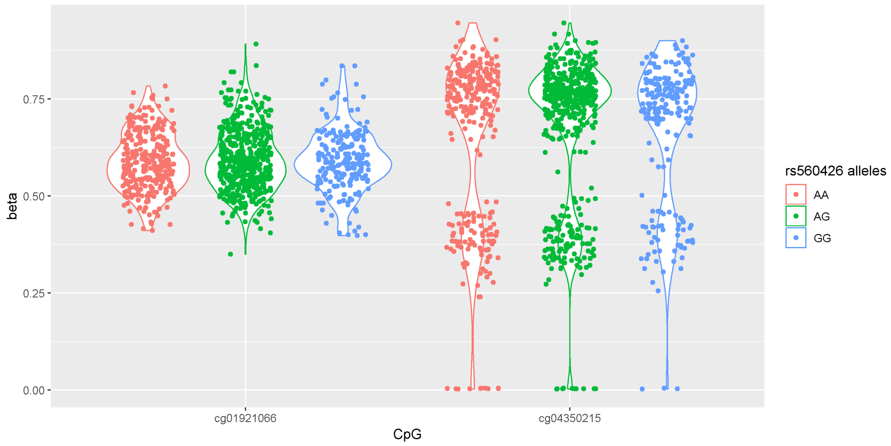

Figure S4: DNA methylation β-values at CpGs found by `gaphunter` to be non-unimodal versus alleles at rs560426 SNP in all our datasets together, shown as dots (each value plotted separately) and as violin plots (distribution).

S2.3 Distributions of the β values in the chosen regions.

The distribution of the averaged β values depends on the number of individuals in each dataset and the number of CpG sites within each category. While the number of categorized CpG sites varied for each SNP (Table 2 in the main text), the averaged β values produced distributions that are mostly normal in shape (Figure 1 in the main text and Figures S5–S8, below). The ranges of these averaged β values are quite narrow, which may be attributed to each measurement being an average value of the actual methylation levels from several cell types in the sample. The underlying β values can be directly translated into a biological effect: $\beta = 0$ means there is no methylation at this CpG site, whereas $\beta = 1$ means this CpG site is fully methylated in all cells.

References

[1] Moreno Uribe, L. M., Fomina, T., Munger, R. G., Romitti, P. A., Jenkins, M. M., Gjessing, H. K., Gjerdevik, M., Christensen, K., Wilcox, A. J., Murray, J. C., et al. (2017). A Population-Based Study of Effects of Genetic Loci on Orofacial Clefts. Journal of Dental Research *96*, 1322–1329.

[2] Xu, Z., Lie, R. T., Wilcox, A. J., Saugstad, O. D., and Taylor, J. A. (2019). A comparison of DNA methylation in newborn blood samples from infants with and without orofacial clefts. Clinical Epigenetics *11*, 40.

[3] Smith, L, M., Baggerly, A, K., Bengtsson, Henrik, Ritchie, E, M., Hansen, and D, K. (2013). illuminaio: An open source IDAT parsing tool for Illumina microarrays. F1000Research *2*.

[4] Xu, Z., Niu, L., Li, L., and Taylor, J. A. (2016). Enmix: a novel background correction method for illumina humanmethylation450 beadchip. Nucleic Acids Research *44*, e20.

[5] Schübeler, D. (2015). Function and information content of DNA methylation. Nature *517*, 321–326.

[6] Jones, P. A. (2012). Functions of DNA methylation: islands, start sites, gene bodies and beyond. Nature Reviews Genetics *13*, 484–492.

[7] Andrews, S. V., Ladd-Acosta, C., Feinberg, A. P., Hansen, K. D., and Fallin, M. D. (2016). ”Gap hunting” to characterize clustered probe signals in Illumina methylation array data. Epigenetics and Chromatin *9*, 1–21.

[8] Hu, K. and Li, J. (2018). Detection and analysis of CpG sites with multimodal DNA methylation level distributions and their relationships with SNPs. BMC Proceedings *12*, 36.

[9] Fortin, J.-P., Triche, T. J., and Hansen, K. D. (2016). Preprocessing, normalization and integration of the Illumina HumanMethylationEPIC array with minfi. Bioinformatics *33*, btw691.

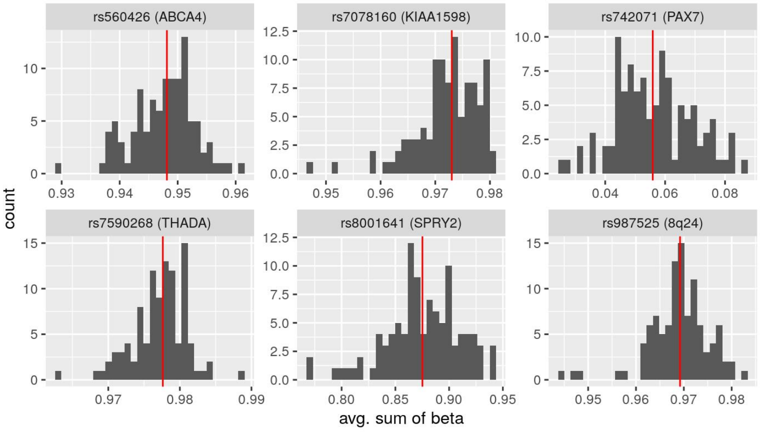

(a) CpG sites from enhancer regions.

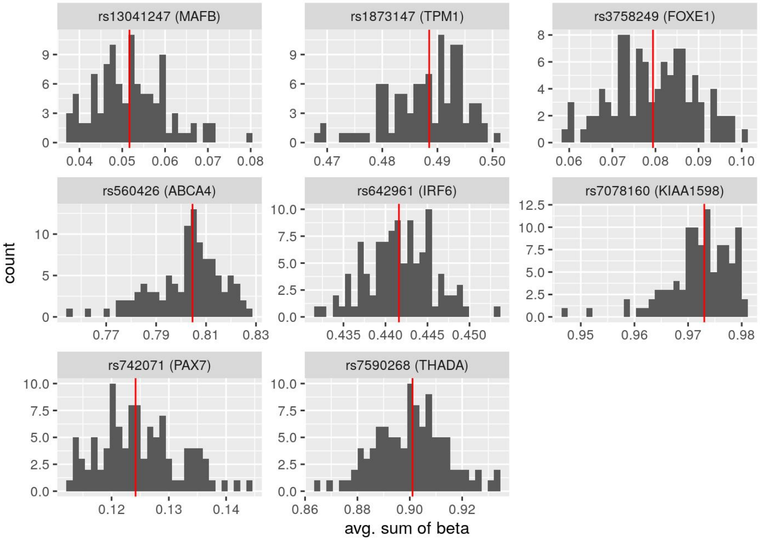

(b) CpG sites from gene regions.

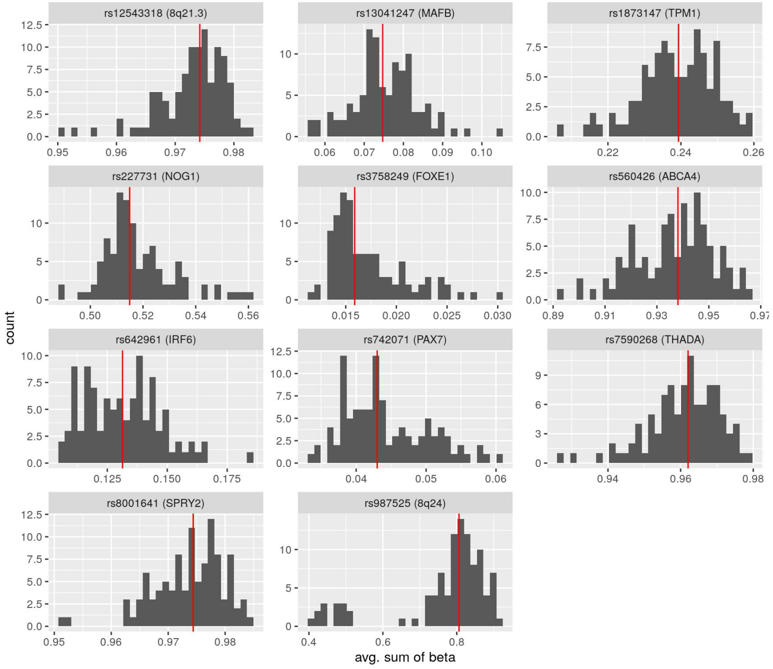

(c) CpG sites from promoter regions.

Figure S5: Histograms for the averaged β values, CLO dataset.

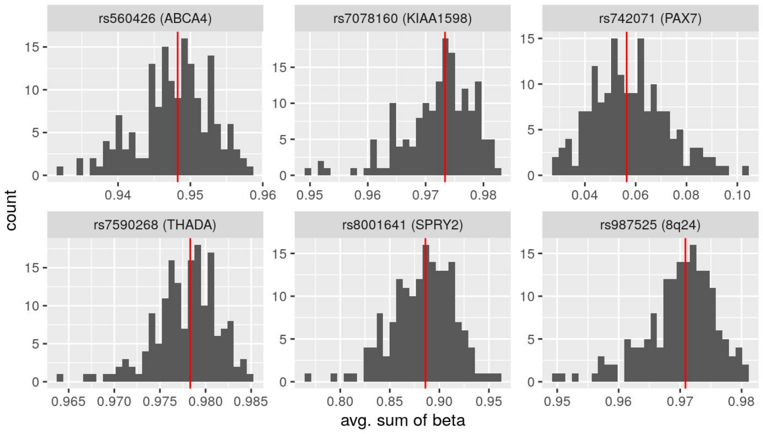

(a) CpG sites from enhancer regions.

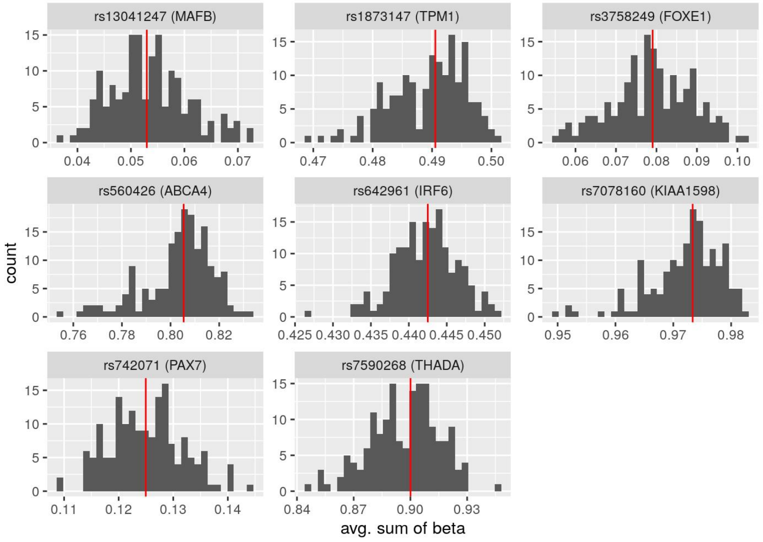

(b) CpG sites from gene regions.

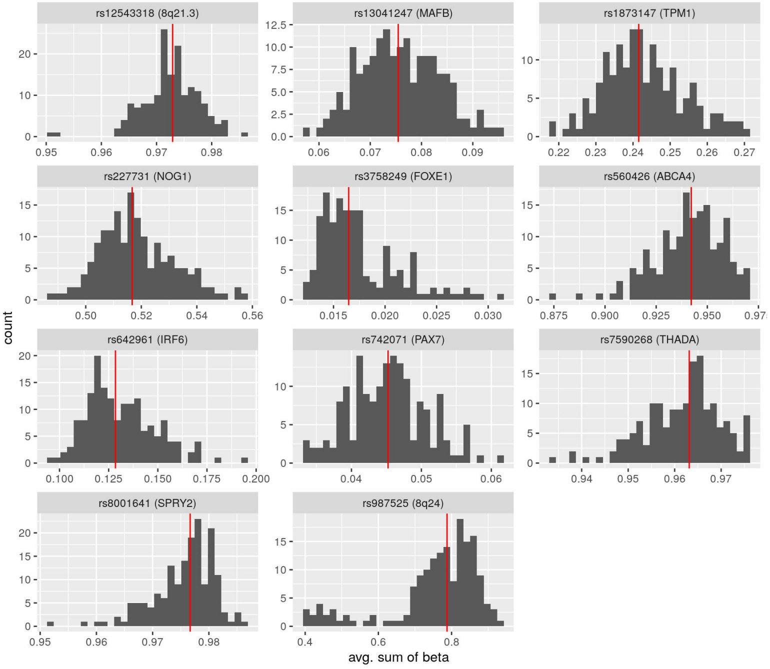

(c) CpG sites from promoter regions.

Figure S6: Histograms for the averaged β values, CLP dataset.

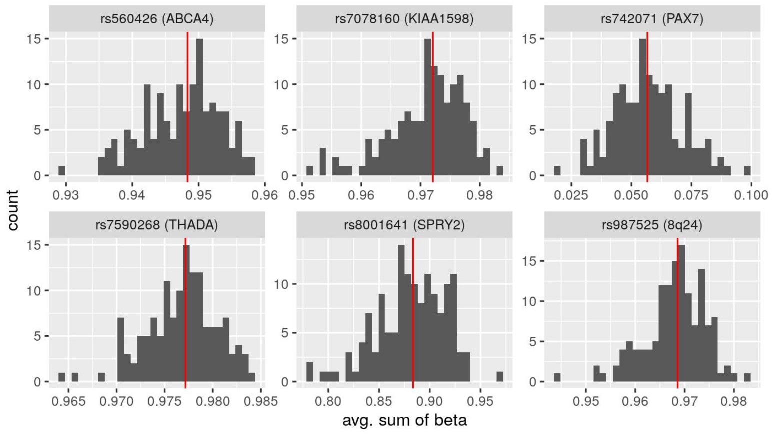

(a) CpG sites from enhancer regions.

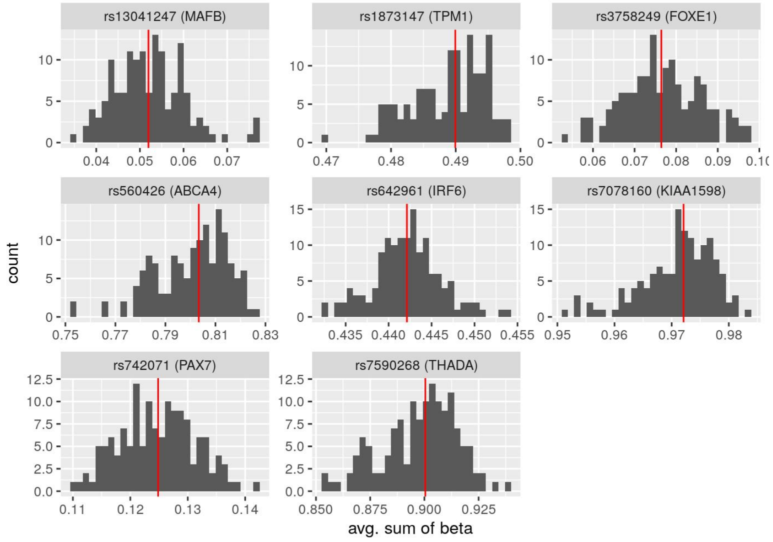

(b) CpG sites from gene regions.

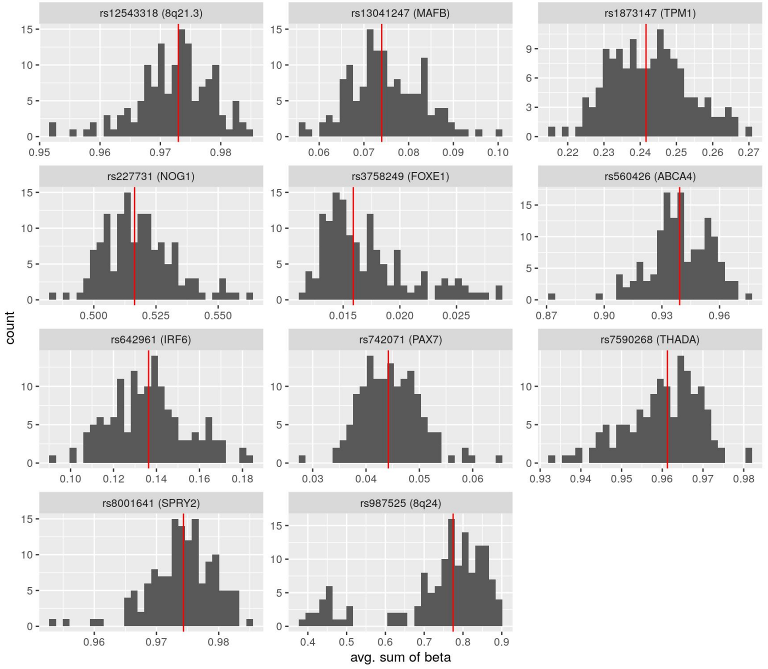

(c) CpG sites from promoter regions.

Figure S7: Histograms for the averaged β values, CPO dataset.

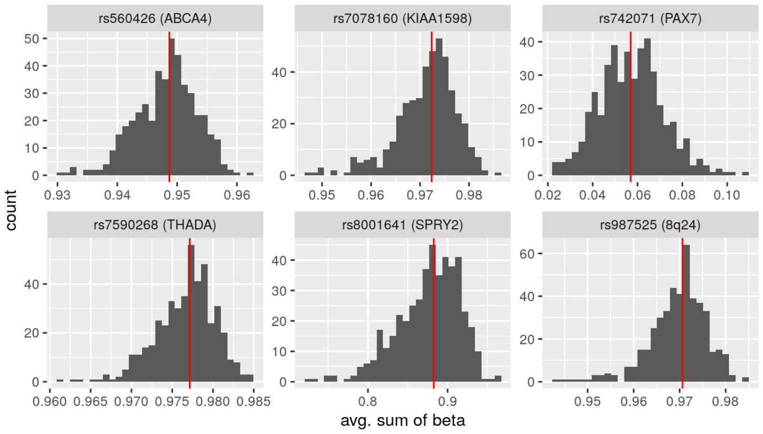

(a) CpG sites from enhancer regions.

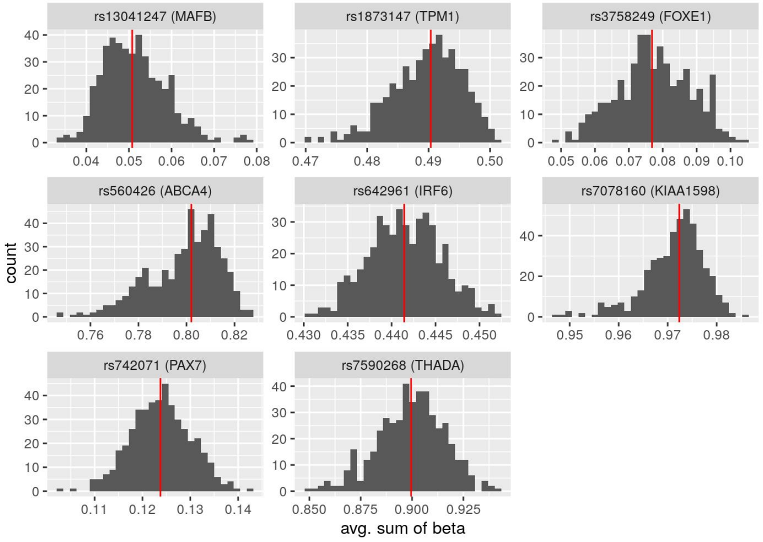

(b) CpG sites from gene regions.

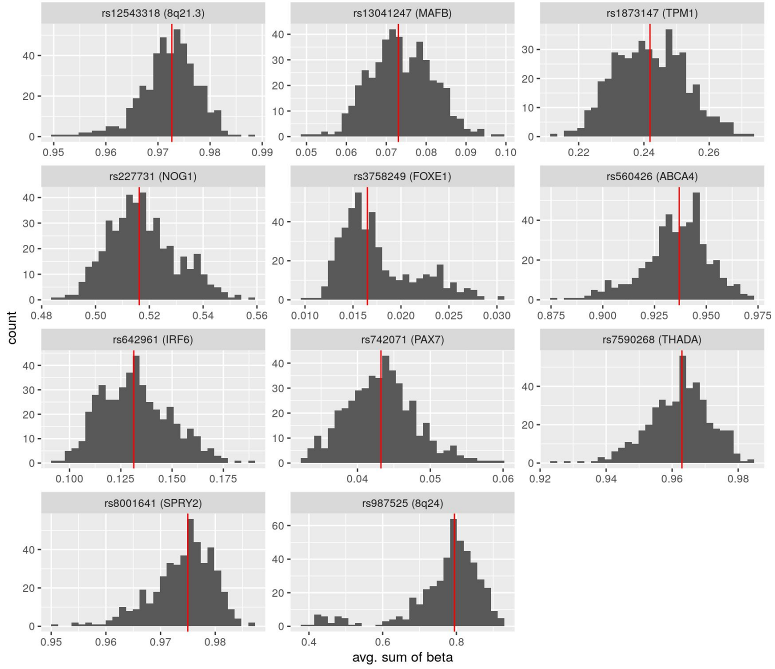

(c) CpG sites from promoter regions.

Figure S8: Histograms for the averaged β values, control dataset.

S3 Main results

Below we provide all the results of the analyses, including p-values for the G×Me, PoO×Me, and trend tests, as well as several figures presenting the relative risk changes for specific SNPs.

S3.1 G×Me

S3.1.1 CL/P dataset

Table S1: The p-values for the G×Me and trend tests, based on the analysis of the CL/P dataset.

	PROMOTER		ENHANCER		GENE	
SNP (gene)	G×Me	trend	G×Me	trend	G×Me	trend
rs12543318 (*8q21.3*)	0.012	0.71	*n.a.*	*n.a.*	*n.a.*	*n.a.*
rs987525 (*8q24*)	0.12	0.057	0.45	0.39	*n.a.*	*n.a.*
rs560426 (*ABCA4*)	0.78	0.59	0.65	0.61	0.12	0.064
rs3758249 (*FOXE1*)	0.65	0.61	*n.a.*	*n.a.*	0.069	0.021
rs642961 (*IRF6*)	0.8	0.82	*n.a.*	*n.a.*	0.59	0.69
rs7078160 (*KIAA1598*	*n.a.*	*n.a.*	0.63	0.48	0.63	0.48
rs13041247 (*MAFB*)	0.88	0.81	*n.a.*	*n.a.*	0.81	0.71
rs227731 (*NOG1*)	0.78	0.7	*n.a.*	*n.a.*	*n.a.*	*n.a.*
rs742071 (*PAX7*)	0.2	0.46	0.59	0.51	0.3	0.15
rs8001641 (*SPRY2*)	0.057	0.022	0.14	0.049	*n.a.*	*n.a.*
rs7590268 (*THADA*)	0.7	0.58	0.49	0.38	0.64	0.94
rs1873147 (*TPM1*)	0.3	0.74	*n.a.*	*n.a.*	0.84	0.72

* *n.a. = not applicable (there were no CpGs belonging to this region around this SNP)*

S3.1.2 CLO dataset

Table S2: The p-values for the G×Me and trend tests, based on the analysis of the CLO dataset.

	PROMOTER		ENHANCER		GENE	
SNP (gene)	G×Me	trend	G×Me	trend	G×Me	trend
rs12543318 (*8q21.3*)	0.32	0.79	*n.a.*	*n.a.*	*n.a.*	*n.a.*
rs987525 (*8q24*)	0.43	0.42	0.41	0.44	*n.a.*	*n.a.*
rs560426 (*ABCA4*)	0.43	0.33	0.91	0.66	0.34	0.19
rs3758249 (*FOXE1*)	0.88	0.61	*n.a.*	*n.a.*	0.8	0.62
rs642961 (*IRF6*)	0.55	0.76	*n.a.*	*n.a.*	0.16	0.96
rs7078160 (*KIAA1598*	*n.a.*	*n.a.*	0.66	0.44	0.66	0.44
rs13041247 (*MAFB*)	0.56	0.45	*n.a.*	*n.a.*	0.92	0.9
rs227731 (*NOG1*)	0.94	0.75	*n.a.*	*n.a.*	*n.a.*	*n.a.*
rs742071 (*PAX7*)	0.064	0.91	0.35	0.52	0.92	0.85
rs8001641 (*SPRY2*)	0.53	0.48	0.63	0.37	*n.a.*	*n.a.*
rs7590268 (*THADA*)	0.17	0.071	0.1	0.15	0.96	0.97
rs1873147 (*TPM1*)	0.4	0.19	*n.a.*	*n.a.*	0.63	0.34

* *n.a. = not applicable (there were no CpGs belonging to this region around this SNP)*

S3.1.3 CLP dataset

Table S3: The p-values for the G×Me and trend tests, based on the analysis of the CLP dataset.

	PROMOTER		ENHANCER		GENE	
SNP (gene)	G×Me	trend	G×Me	trend	G×Me	trend
rs12543318 (*8q21.3*)	0.29	0.69	*n.a.*	*n.a.*	*n.a.*	*n.a.*
rs987525 (*8q24*)	0.21	0.094	0.54	0.6	*n.a.*	*n.a.*
rs560426 (*ABCA4*)	0.25	0.16	0.43	0.65	0.69	0.4
rs3758249 (*FOXE1*)	0.7	0.46	*n.a.*	*n.a.*	0.001	0.00028
rs642961 (*IRF6*)	0.81	0.69	*n.a.*	*n.a.*	0.89	0.65
rs7078160 (*KIAA1598*	*n.a.*	*n.a.*	0.44	0.44	0.44	0.44
rs13041247 (*MAFB*)	0.96	0.8	*n.a.*	*n.a.*	0.84	0.57
rs227731 (*NOG1*)	0.79	0.57	*n.a.*	*n.a.*	*n.a.*	*n.a.*
rs742071 (*PAX7*)	0.28	0.17	0.5	0.39	0.19	0.098
rs8001641 (*SPRY2*)	0.22	0.081	0.25	0.12	*n.a.*	*n.a.*
rs7590268 (*THADA*)	0.63	0.37	0.9	0.74	0.98	0.84
rs1873147 (*TPM1*)	0.23	0.8	*n.a.*	*n.a.*	0.39	0.32

* *n.a. = not applicable (there were no CpGs belonging to this region around this SNP)*

S3.1.4 CPO dataset

Table S4: The p-values for the G×Me and trend tests, based on the analysis of the CPO dataset.

	PROMOTER		ENHANCER		GENE	
SNP (gene)	G×Me	trend	G×Me	trend	G×Me	trend
rs12543318 (*8q21.3*)	0.056	0.12	*n.a.*	*n.a.*	*n.a.*	*n.a.*
rs987525 (*8q24*)	0.036	0.061	0.88	0.63	*n.a.*	*n.a.*
rs560426 (*ABCA4*)	0.51	0.62	0.42	0.19	0.37	0.17
rs3758249 (*FOXE1*)	0.9	0.77	*n.a.*	*n.a.*	0.076	0.026
rs642961 (*IRF6*)	0.8	0.91	*n.a.*	*n.a.*	0.98	0.93
rs7078160 (*KIAA1598*	*n.a.*	*n.a.*	0.86	0.75	0.86	0.75
rs13041247 (*MAFB*)	0.68	0.77	*n.a.*	*n.a.*	0.46	0.51
rs227731 (*NOG1*)	0.13	0.74	*n.a.*	*n.a.*	*n.a.*	*n.a.*
rs742071 (*PAX7*)	0.54	0.3	0.56	0.5	0.49	0.26
rs8001641 (*SPRY2*)	0.36	0.49	0.49	0.59	*n.a.*	*n.a.*
rs7590268 (*THADA*)	0.44	0.35	0.79	0.49	0.83	0.71
rs1873147 (*TPM1*)	0.83	0.72	*n.a.*	*n.a.*	0.76	0.87

* *n.a. = not applicable (there were no CpGs belonging to this region around this SNP)*

S3.1.5 Control dataset

Table S5: The p-values for the G×Me and trend tests, based on the analysis of the control dataset.

	PROMOTER		ENHANCER		GENE	
SNP (gene)	G×Me	trend	G×Me	trend	G×Me	trend
rs12543318 (*8q21.3*)	0.15	0.12	*n.a.*	*n.a.*	*n.a.*	*n.a.*
rs987525 (*8q24*)	0.048	0.019	0.22	0.33	*n.a.*	*n.a.*
rs560426 (*ABCA4*)	0.83	0.59	0.16	0.53	0.03	0.047
rs3758249 (*FOXE1*)	0.59	0.8	*n.a.*	*n.a.*	8.6e-05	8.7e-05
rs642961 (*IRF6*)	0.12	0.82	*n.a.*	*n.a.*	0.38	0.75
rs7078160 (*KIAA1598*	*n.a.*	*n.a.*	0.25	0.15	0.25	0.15
rs13041247 (*MAFB*)	0.27	0.86	*n.a.*	*n.a.*	0.88	0.62
rs227731 (*NOG1*)	0.57	0.6	*n.a.*	*n.a.*	*n.a.*	*n.a.*
rs742071 (*PAX7*)	0.72	0.47	0.6	0.31	0.33	0.57
rs8001641 (*SPRY2*)	0.32	0.84	0.84	0.59	*n.a.*	*n.a.*
rs7590268 (*THADA*)	0.54	0.27	0.85	0.68	0.46	0.64
rs1873147 (*TPM1*)	5.6e-05	1.2e-05	*n.a.*	*n.a.*	0.41	0.18

* *n.a. = not applicable (there were no CpGs belonging to this region around this SNP)*

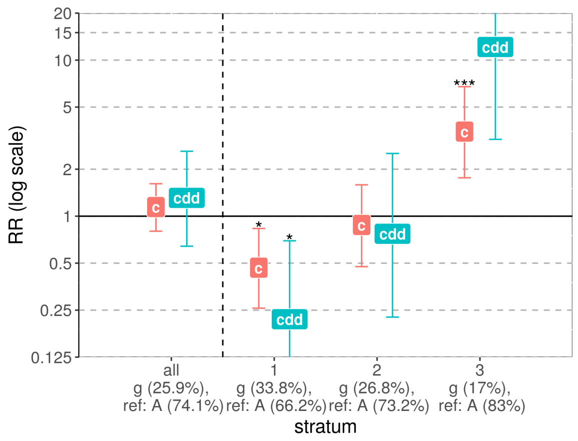

Figure S9: G×Me effect between rs1873147 in *TPM1* and the methylation level at the CpGs from the gene region, showing the top result from the analysis of the control dataset. The x-axis groups the results into the unstratified dataset "all" and the results for each stratum: "1" denoting low methylation level, "2" — medium, and "3" — high methylation level. The minor and reference alleles are given for each group, along with their frequencies. The y-axis shows the relative risk on a log scale, with "c" denoting the child effect when only one minor allele is inherited (single dose), and "cdd" denoting the child effect when two such alleles are inherited (double dose).

S3.1.6 Randomly chosen SNPs, CL/P dataset

Table S6: The 20 randomly chosen SNPs and their chromosomal positions.

SNP NAME	POSITION
rs6659735	chr1:18983697
rs2867125	chr2:622827
rs713586	chr2:25158008
rs4621895	chr9:20582556
rs10818094	chr9:100563828
rs1443433	chr9:100579219
rs10984103	chr9:100639275
rs4752566	chr10:123267631
rs2912771	chr10:123299297
rs2981428	chr10:123329429
rs3750817	chr10:123332577
rs10767664	chr11:27725986
rs7138803	chr12:50247468
rs9939609	chr16:53820527
rs1546124	chr16:84872051
rs17760296	chr17:54615617
rs633265	chr18:57831468
rs17782313	chr18:57851097
rs6010718	chr20:62719376
rs3752462	chr22:36710183

S3.2 PoO×Me

S3.2.1 CL/P dataset

Table S7: The p-values for the PoO×Me and trend tests, based on the analysis of the CL/P dataset.

	PROMOTER		ENHANCER		GENE	
SNP (gene)	PoO×Me	trend	PoO×Me	trend	PoO×Me	trend
rs12543318 (*8q21.3*)	0.42	0.81	*n.a.*	*n.a.*	*n.a.*	*n.a.*
rs987525 (*8q24*)	0.058	0.35	0.45	0.39	*n.a.*	*n.a.*
rs560426 (*ABCA4*)	0.76	0.63	0.65	0.61	0.12	0.064
rs3758249 (*FOXE1*)	0.059	0.023	*n.a.*	*n.a.*	0.069	0.021
rs642961 (*IRF6*)	0.34	0.17	*n.a.*	*n.a.*	0.59	0.69
rs7078160 (*KIAA1598*)	*n.a.*	*n.a.*	0.63	0.48	0.63	0.48
rs13041247 (*MAFB*)	0.11	0.43	*n.a.*	*n.a.*	0.81	0.71
rs227731 (*NOG1*)	0.0032	0.36	*n.a.*	*n.a.*	*n.a.*	*n.a.*
rs742071 (*PAX7*)	0.49	0.53	0.59	0.51	0.3	0.15
rs8001641 (*SPRY2*)	0.34	0.68	0.14	0.049	*n.a.*	*n.a.*
rs7590268 (*THADA*)	0.44	0.21	0.49	0.38	0.64	0.94
rs1873147 (*TPM1*)	0.13	0.45	*n.a.*	*n.a.*	0.84	0.72

* *n.a.* = not applicable (there were no CpGs belonging to this region around this SNP)

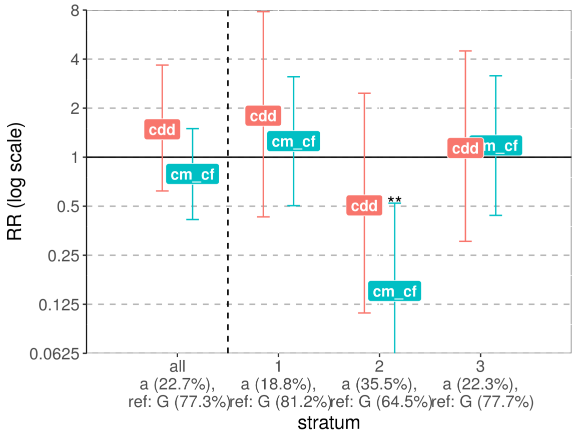

(a) CpGs from the gene region near rs642961 in *IRF6*.

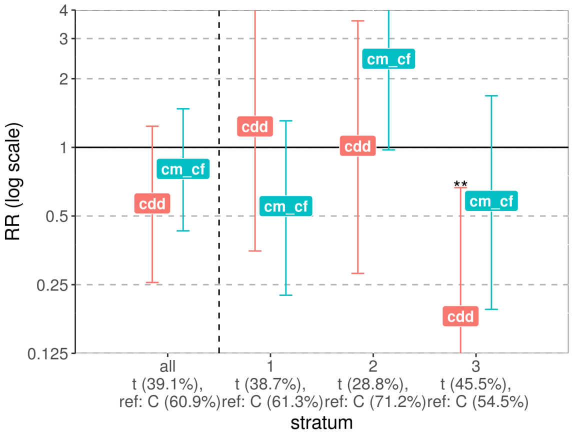

(b) CpGs from the gene region near rs3758249 in *FOXE1*.

Figure S10: PoO×Me interaction between a parent-of-origin effect of a SNP and the methylation level of the CpGs nearby; top results from the analysis of the CL/P dataset. The x-axis groups the results into the unstratified dataset "all" and the results for each stratum: "1" denoting low methylation level, "2" — medium, and "3" — high methylation level. The minor and reference alleles are given for each group, along with their frequencies. The y-axis shows the relative risk on a log scale, with "cm_cf" denoting the parent-of-origin effect when only one minor allele is inherited (single dose), and "cdd" denoting the parent-of-origin effect when two such alleles are inherited (double dose).

S3.2.2 CLO dataset

Table S8: The p-values for the PoO×Me and trend tests, based on the analysis of the CLO dataset.

	PROMOTER		ENHANCER		GENE	
SNP (gene)	PoO×Me	trend	PoO×Me	trend	PoO×Me	trend
rs12543318 (*8q21.3*)	0.024	0.62	*n.a.*	*n.a.*	*n.a.*	*n.a.*
rs987525 (*8q24*)	0.087	0.044	0.41	0.44	*n.a.*	*n.a.*
rs560426 (*ABCA4*)	0.72	0.54	0.91	0.66	0.34	0.19
rs3758249 (*FOXE1*)	0.62	0.33	*n.a.*	*n.a.*	0.8	0.62
rs642961 (*IRF6*)	0.1	0.13	*n.a.*	*n.a.*	0.16	0.96
rs7078160 (*KIAA1598*	*n.a.*	*n.a.*	0.66	0.44	0.66	0.44
rs13041247 (*MAFB*)	0.32	0.16	*n.a.*	*n.a.*	0.92	0.9
rs227731 (*NOG1*)	0.012	0.026	*n.a.*	*n.a.*	*n.a.*	*n.a.*
rs742071 (*PAX7*)	0.93	0.71	0.35	0.52	0.92	0.85
rs8001641 (*SPRY2*)	0.15	0.36	0.63	0.37	*n.a.*	*n.a.*
rs7590268 (*THADA*)	0.6	0.31	0.1	0.15	0.96	0.97
rs1873147 (*TPM1*)	0.32	0.99	*n.a.*	*n.a.*	0.63	0.34

* *n.a. = not applicable (there were no CpGs belonging to this region around this SNP)*

S3.2.3 CLP dataset

Table S9: The p-values for the PoO×Me and trend tests, based on the analysis of the CLP dataset.

	PROMOTER		ENHANCER		GENE	
SNP (gene)	PoO×Me	trend	PoO×Me	trend	PoO×Me	trend
rs12543318 (*8q21.3*)	0.57	0.58	*n.a.*	*n.a.*	*n.a.*	*n.a.*
rs987525 (*8q24*)	0.0037	0.087	0.54	0.6	*n.a.*	*n.a.*
rs560426 (*ABCA4*)	0.99	0.87	0.43	0.65	0.69	0.4
rs3758249 (*FOXE1*)	0.27	0.2	*n.a.*	*n.a.*	0.001	0.00028
rs642961 (*IRF6*)	0.049	0.21	*n.a.*	*n.a.*	0.89	0.65
rs7078160 (*KIAA1598*	*n.a.*	*n.a.*	0.44	0.44	0.44	0.44
rs13041247 (*MAFB*)	0.11	0.66	*n.a.*	*n.a.*	0.84	0.57
rs227731 (*NOG1*)	0.0093	0.027	*n.a.*	*n.a.*	*n.a.*	*n.a.*
rs742071 (*PAX7*)	0.17	0.91	0.5	0.39	0.19	0.098
rs8001641 (*SPRY2*)	0.88	0.76	0.25	0.12	*n.a.*	*n.a.*
rs7590268 (*THADA*)	0.77	0.56	0.9	0.74	0.98	0.84
rs1873147 (*TPM1*)	0.33	0.51	*n.a.*	*n.a.*	0.39	0.32

* *n.a. = not applicable (there were no CpGs belonging to this region around this SNP)*

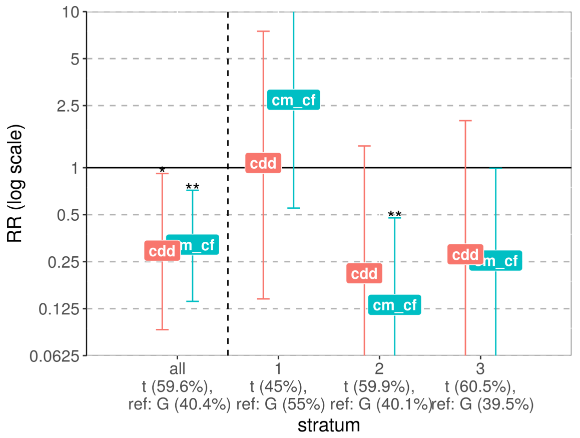

(a) CpGs from the promoter region near rs227731 in *NOG1*.

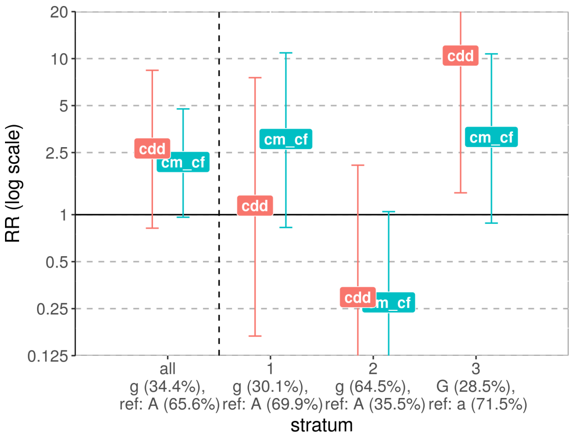

(b) CpGs from the gene region near rs560426 in *ABCA4*.

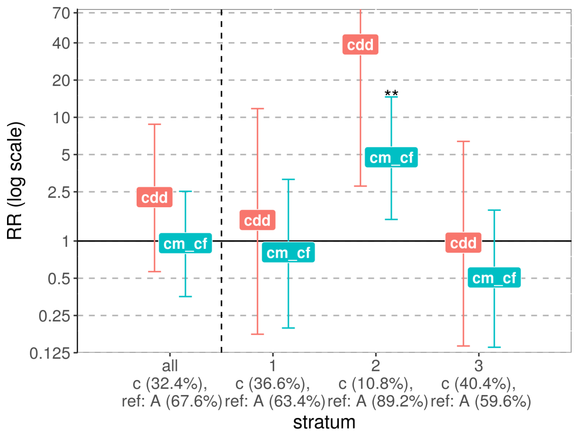

(c) CpGs from the promoter region near rs12543318 in *8q21.3*.

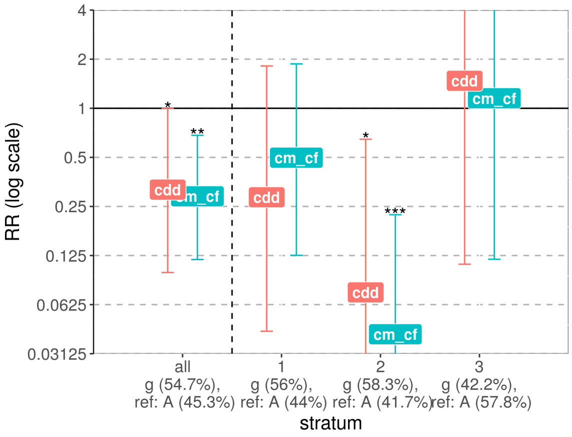

(d) CpGs from the enhancer region near rs8001641 in *SPRY2*.

Figure S11: PoO×Me interaction between a parent-of-origin effect of a SNP and the methylation level of the CpGs nearby; top results from the analysis of the CLO dataset. The x-axis groups the results into the unstratified dataset "all" and the results for each stratum: "1" denoting low methylation level, "2" — medium, and "3" — high methylation level. The minor and reference alleles are given for each group, along with their frequencies. The y-axis shows the relative risk on a log scale, with "cm_cf" denoting the parent-of-origin effect when only one minor allele is inherited (single dose), and "cdd" denoting the parent-of-origin effect when two such alleles are inherited (double dose).

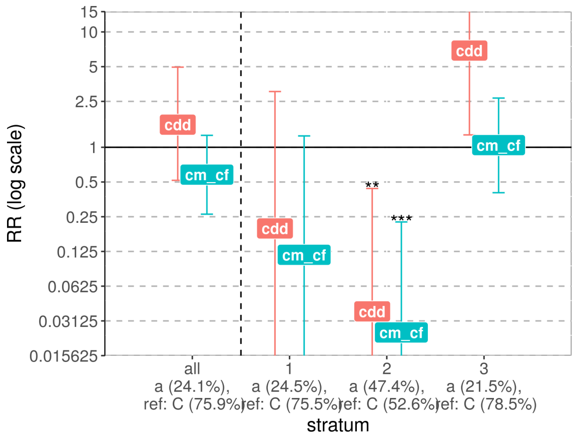

(a) CpGs from the promoter region near rs987525 in *8q24*.

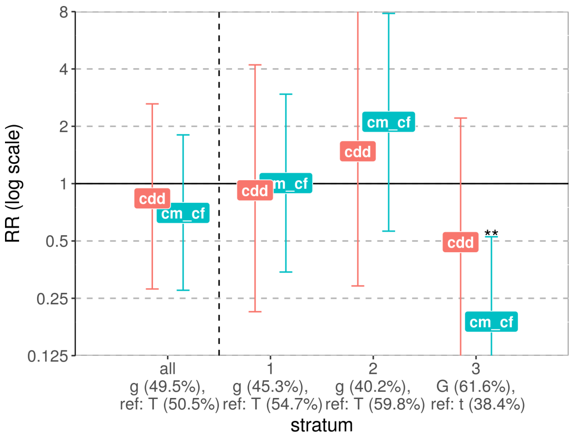

(b) CpGs from the promoter region near rs227731 in *NOG1*.

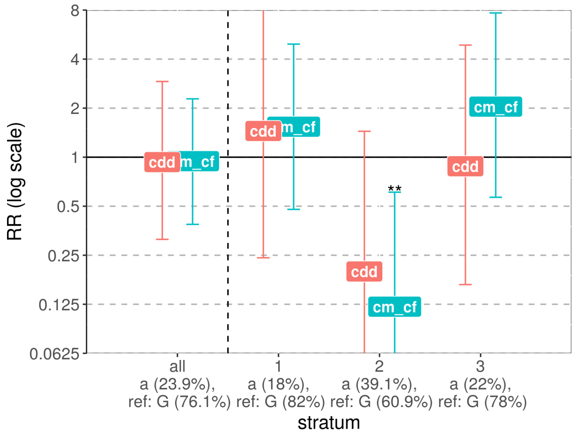

(c) CpGs from the gene region near rs642961 in *IRF6*.

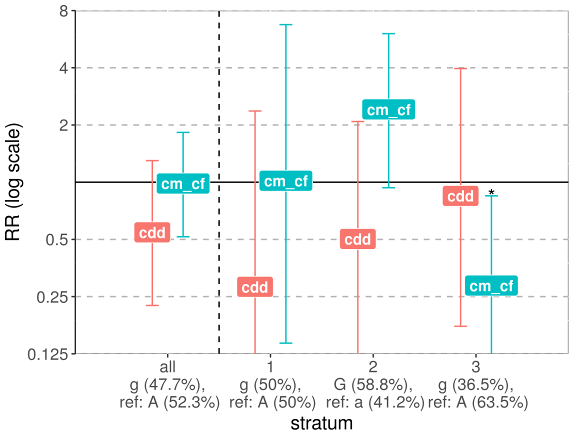

(d) CpGs from the enhancer region near rs8001641 in *SPRY2*.

Figure S12: PoO×Me interaction between a parent-of-origin effect of a SNP and the methylation level of the CpGs nearby; top results from the analysis of the CLP dataset. The x-axis groups the results into the unstratified dataset “all” and the results for each stratum: “1” denoting low methylation level, “2” — medium, and “3” — high methylation level. The minor and reference alleles are given for each group, along with their frequencies. The y-axis shows the relative risk on a log scale, with “cm_cf” denoting the parent-of-origin effect when only one minor allele is inherited (single dose), and “cdd” denoting the parent-of-origin effect when two such alleles are inherited (double dose).

S3.2.4 CPO dataset

Table S10: The p-values for the PoO×Me and trend tests, based on the analysis of the CPO dataset.

	PROMOTER		ENHANCER		GENE	
SNP (gene)	PoO×Me	trend	PoO×Me	trend	PoO×Me	trend
rs12543318 (*8q21.3*)	0.47	0.22	*n.a.*	*n.a.*	*n.a.*	*n.a.*
rs987525 (*8q24*)	0.31	0.18	0.88	0.63	*n.a.*	*n.a.*
rs560426 (*ABCA4*)	0.57	0.41	0.42	0.19	0.37	0.17
rs3758249 (*FOXE1*)	0.97	0.79	*n.a.*	*n.a.*	0.076	0.026
rs642961 (*IRF6*)	0.86	0.82	*n.a.*	*n.a.*	0.98	0.93
rs7078160 (*KIAA1598*	*n.a.*	*n.a.*	0.86	0.75	0.86	0.75
rs13041247 (*MAFB*)	0.024	0.025	*n.a.*	*n.a.*	0.46	0.51
rs227731 (*NOG1*)	0.36	0.96	*n.a.*	*n.a.*	*n.a.*	*n.a.*
rs742071 (*PAX7*)	0.17	0.74	0.56	0.5	0.49	0.26
rs8001641 (*SPRY2*)	0.21	0.29	0.49	0.59	*n.a.*	*n.a.*
rs7590268 (*THADA*)	0.32	0.3	0.79	0.49	0.83	0.71
rs1873147 (*TPM1*)	0.2	0.21	*n.a.*	*n.a.*	0.76	0.87

* *n.a. = not applicable (there were no CpGs belonging to this region around this SNP)*

S3.2.5 Control dataset

Table S11: The p-values for the PoO×Me and trend tests, based on the analysis of the control dataset.

	PROMOTER		ENHANCER		GENE	
SNP (gene)	PoO×Me	trend	PoO×Me	trend	PoO×Me	trend
rs12543318 (*8q21.3*)	0.0056	0.002	*n.a.*	*n.a.*	*n.a.*	*n.a.*
rs987525 (*8q24*)	0.08	0.98	0.22	0.33	*n.a.*	*n.a.*
rs560426 (*ABCA4*)	0.1	0.3	0.16	0.53	0.03	0.047
rs3758249 (*FOXE1*)	0.61	0.71	*n.a.*	*n.a.*	8.6e-05	8.7e-05
rs642961 (*IRF6*)	0.39	0.21	*n.a.*	*n.a.*	0.38	0.75
rs7078160 (*KIAA1598*	*n.a.*	*n.a.*	0.25	0.15	0.25	0.15
rs13041247 (*MAFB*)	0.98	0.98	*n.a.*	*n.a.*	0.88	0.62
rs227731 (*NOG1*)	0.49	0.57	*n.a.*	*n.a.*	*n.a.*	*n.a.*
rs742071 (*PAX7*)	0.41	0.82	0.6	0.31	0.33	0.57
rs8001641 (*SPRY2*)	0.63	0.71	0.84	0.59	*n.a.*	*n.a.*
rs7590268 (*THADA*)	0.49	0.65	0.85	0.68	0.46	0.64
rs1873147 (*TPM1*)	0.91	0.99	*n.a.*	*n.a.*	0.41	0.18

* *n.a. = not applicable (there were no CpGs belonging to this region around this SNP)*

S3.2.6 PoO scan, CLP dataset

Table S12: Top 20 SNPs from the PoO scan of the CLP dataset.

SNP NAME	POSITION	RRR (95% CI)	p-VALUE
rs29941	chr19:34309532	0.57 (0.4, 0.82)	2.1e-03
rs2235371	chr1:209964080	2.4 (1.3, 4.3)	6.2e-03
rs4752028	chr10:118834991	0.71 (0.52, 0.96)	2.4e-02
rs633265	chr18:57831468	0.72 (0.52, 0.99)	4.7e-02
rs2912760	chr10:123271059	0.73 (0.53, 1)	5.2e-02
rs11084753	chr19:34322137	0.73 (0.51, 1)	8.1e-02
rs8001641	chr13:80692811	1.3 (0.97, 1.7)	8.1e-02
rs2912771	chr10:123299297	0.76 (0.55, 1)	8.6e-02
rs1002246	chr22:36714659	1.3 (0.94, 1.7)	1.2e-01
rs6701037	chr1:175120079	0.79 (0.59, 1.1)	1.3e-01
rs7078160	chr10:118827560	0.81 (0.61, 1.1)	1.4e-01
rs766325	chr1:18956458	1.3 (0.93, 1.7)	1.5e-01
rs4621895	chr9:20582556	1.2 (0.91, 1.7)	1.8e-01
rs470563	chr18:74671441	0.81 (0.59, 1.1)	1.9e-01
rs7138803	chr12:50247468	0.82 (0.59, 1.1)	2.2e-01
rs987525	chr8:129946154	0.84 (0.65, 1.1)	2.3e-01
rs3752075	chr18:74637067	1.2 (0.89, 1.6)	2.3e-01
rs17782313	chr18:57851097	0.84 (0.64, 1.1)	2.4e-01
rs7864322	chr9:100548934	0.82 (0.59, 1.2)	2.5e-01
rs571312	chr18:57839769	0.85 (0.65, 1.1)	2.7e-01

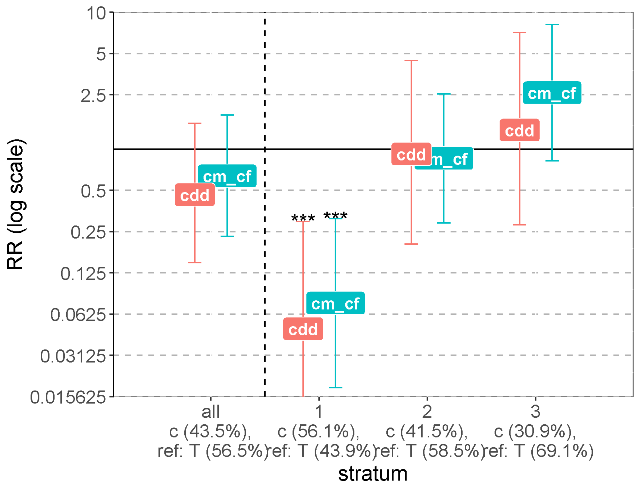

Figure S13: PoO×Me interaction between a parent-of-origin effect of rs766325 and the methylation level of the CpGs from the promoter region nearby; top result from the PoO scan of the CLP dataset. The x-axis groups the results into the unstratified dataset "all" and the results for each stratum: "1" denoting low methylation level, "2" — medium, and "3" — high methylation level. The minor and reference alleles are given for each group, along with their frequencies. The y-axis shows the relative risk on a log scale, with "cm_cf" denoting the parent-of-origin effect when only one minor allele is inherited (single dose), and "cdd" denoting the parent-of-origin effect when two such alleles are inherited (double dose).

S3.2.7 Checking the significant PoO×Me interaction promoter—rs227731

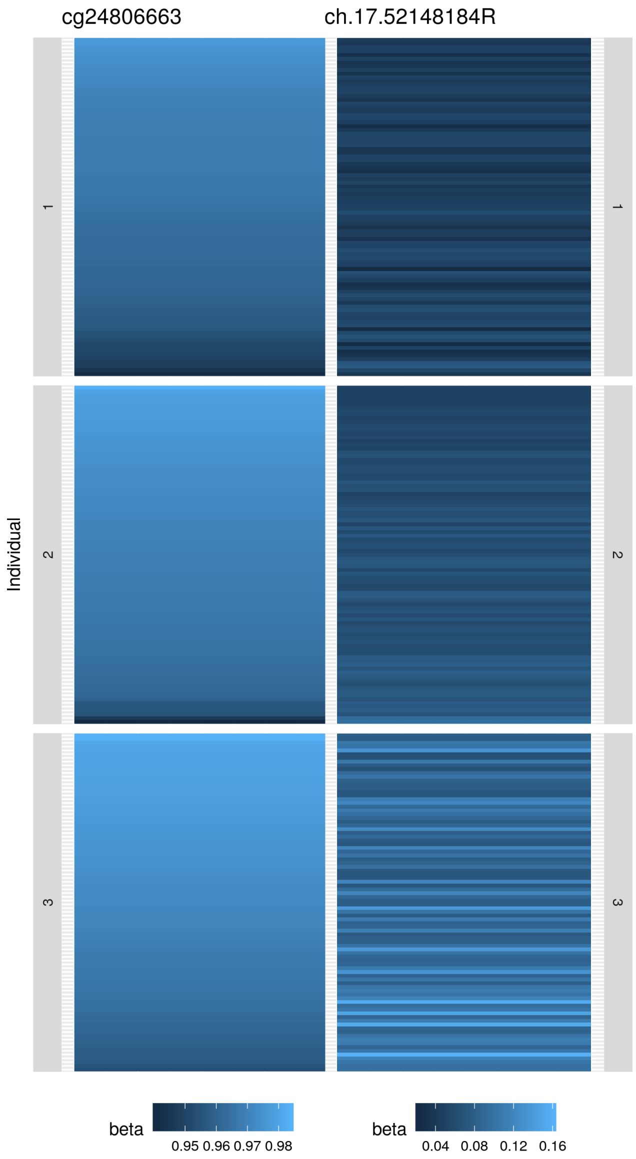

Figure S14: Individual β-values of the two CpGs within a promoter region near rs227731 (*NOG1*). Each row represents an individual, each CpG has its own shading scheme, as depicted at the bottom of the figure. The rows are grouped by the stratum they belong to (1 through 3), which was calculated based on an average β-value of the two CpGs. The rows are ordered by β-value of the left CpG.

Table S13: The p-values for the PoO$\times$Me and trend tests, based on the analysis of rs227731 in *NOG1* and DNA methylation β-values of only ch.17.52148184R from the promoter region nearby.

	p-value	
OFC subtype	PoO$\times$Me	trend
CLO	0.16	0.20
CLP	0.0094	0.099
CL/P	0.021	0.78

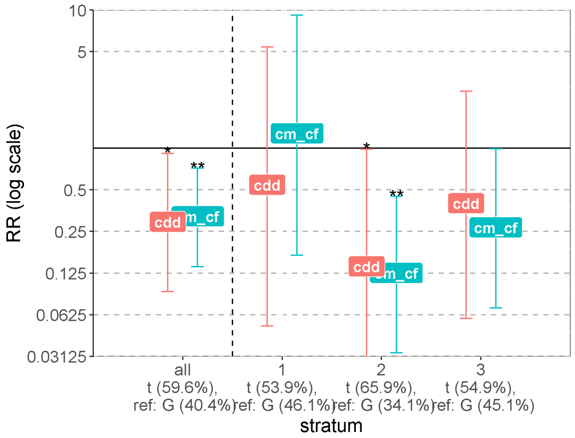

(a) CLO dataset

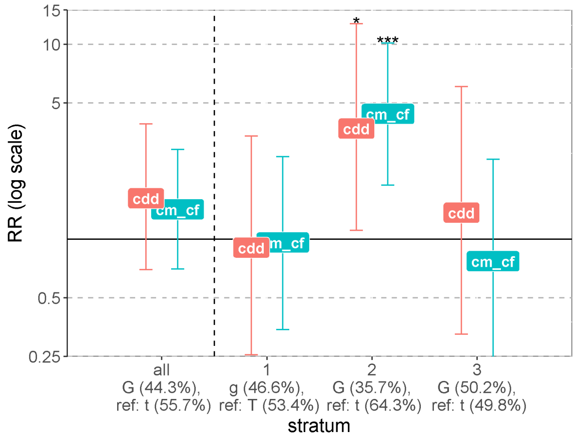

(b) CL/P dataset

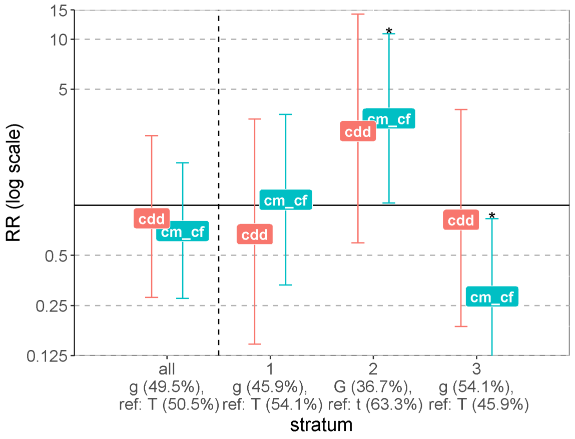

(c) CLP dataset

Figure S15: PoO$\times$Me effect between rs227731 in *NOG1* and the methylation of one CpG from the promoter region nearby, i.e., ch.17.52148184R. The x-axis groups the results into the unstratified dataset "all" and the results for each stratum: "1" denoting low methylation level, "2" — medium, and "3" — high methylation level. The minor and reference alleles are given for each group, along with their frequencies. The y-axis shows the relative risk on a log scale, with "cm_cf" denoting the parent-of-origin effect when only one minor allele is inherited (single dose), and "cdd" denoting the parent-of-origin effect when two such alleles are inherited (double dose).

S3.2.8 Transcription factors binding to CpGs within promoter region near rs227731

Table S14: Transcription factors likely binding to the active cytosine within ch.17.52148184R — one of the two CpGs within promoter region near rs227731, based on the data from the JASPAR database.

PROTEIN NAME	UNIPROT ID	FUNCTION SUMMARY*
NR2C2(var.2)	P49116	"Orphan nuclear receptor that can act as a repressor or activator of transcription. An important repressor of nuclear receptor signaling pathways such as retinoic acid receptor, retinoid X, vitamin D3 receptor, thyroid hormone receptor and estrogen receptor pathways. [...] Plays a fundamental role in early embryonic development and embryonic stem cells. Required for normal spermatogenesis and cerebellum development."
BARX1	Q9HBU1	"Transcription factor, which is involved in craniofacial development, in odontogenesis and in stomach organogenesis."
SCRT2	Q9NQ03	unprecise description that includes more than 3 distinct TFs, may be involved in transciptional regulation
GRLH2	Q6ISB3	"important role in primary neurulation and in epithelial development"
DMRTA2	Q96SC8	"May be involved in sexual development."
VDR	P11473	"Nuclear receptor for calcitriol, the active form of vitamin D3 which mediates the action of this vitamin on cells"
SNAI1	O95863	"Involved in induction of the epithelial to mesenchymal transition (EMT), formation and maintenance of embryonic mesoderm, growth arrest, survival and cell migration."
SNAI3	Q3KNW1	"Seems to inhibit myoblast differentiation."
HMBOX1	Q6NT76	"positively regulates telomere elongation"
HOXB3	P14651	"Sequence-specific transcription factor which is part of a developmental regulatory system that provides cells with specific positional identities on the anterior-posterior axis."
MIXL1	Q9H2W2	"Transcription factor that play a central role in proper axial mesendoderm morphogenesis and endoderm formation."
BSX	Q3C1V8	"DNA binding protein that function as transcriptional activator. Is essential for normal postnatal growth and nursing."
DLX5	P56178	"Transcriptional factor involved in bone development."
DRGX	A6NNA5	"Transcription factor required for the formation of correct projections from nociceptive sensory neurons to the dorsal horn of the spinal cord and normal perception of pain."
HOXA4	Q00056	"Sequence-specific transcription factor which is part of a developmental regulatory system that provides cells with specific positional identities on the anterior-posterior axis."
HOXA7	P31268	as above
HOXB8	P17481	as above
HOXC4	P09017	as above
HOXD4	P09016	as above

* taken from UniProt webpages

Table S15: Transcription factors likely binding to the active cytosine within cg24806663 — on of the two CpGs within promoter region near rs227731, based on the data from the JASPAR database.

PROTEIN NAME	UNIPROT ID	FUNCTION SUMMARY*
TCFL5	Q9UL49	not clear whether this is a TF
NR1D2	Q14995	"Transcriptional repressor which coordinates circadian rhythm and metabolic pathways in a heme-dependent manner."
NR2C2	P49116	"Orphan nuclear receptor that can act as a repressor or activator of transcription."
ZNF274	Q96GC6	"Probable transcription repressor."

* taken from UniProt webpages
