# Supplementary material for: Gene–methylation interactions: discovering region-wise DNA methylation levels that modify SNP-associated disease risk
Source: Clin Epigenetics. 2020 Jul 16;12:109. doi: 10.1186/s13148-020-00881-x (PMC7367265; doi:10.1186/s13148-020-00881-x)
Supplement: Supplementary file 2 — Additional file 2 Details of the statistical methods. [file 13148_2020_881_MOESM2_ESM.pdf]

Gene-methylation interactions: Discovering region-wise DNA methylation levels that modify SNP-associated disease risk

Julia Romanowska[1,2,4], Øystein A. Haaland[1], Astanand Jugessur[1,3,4], Miriam Gjerdevik[1,3], Zongli Xu[5], Jack Taylor[5], Allen J. Wilcox[5], Inge Jonassen[2], Rolv Terje Lie[1,4], Håkon K. Gjessing[1,4]

[1] *Department of Global Public Health and Primary Care, University of Bergen, Bergen, N-5020, Norway;* [2] *Computational Biology Unit, University of Bergen, Bergen, N-5020, Norway;* [3] *Department of Genetics and Bioinformatics, Norwegian Institute of Public Health, Oslo, N-0473, Norway;* [4] *Centre for Fertility and Health, Norwegian Institute of Public Health, Oslo, N-0213, Norway;* [5] *National Institute of Environmental Health Sciences, Research Triangle Park, NC 27709, USA*

Additional File 2

Statistical methods — details

As we have shown previously and in the main text, we assume the penetrance model

$$P(D|C_{ij}, Me = m) = B^{(m)} \cdot RR_{M,i}^{(m)} \cdot RR_{F,j}^{(m)}, \tag{S.1}$$

And, since for case triads,

$$P(M, F, C, Me|D) = \frac{P(D|M, F, C, Me)P(Me|M, F, C)P(C|M, F)P(M, F)}{P(D)}, \tag{S.2}$$

The factor $P(Me|M, F, C)$, i.e. the population distribution of methylation within genetic strata, is crucial in our analyses. When independent control triads are available, we make an assumption – for simplicity – of rare disease. In that case,

$$P(M, F, C, Me|\bar{D}) \approx P(Me|M, F, C)P(C|M, F)P(M, F). \tag{S.3}$$

Thus, when controls are available, there is no need to make specific assumptions about $P(Me|M, F, C)$ since it can be estimated from control triads. With only case-parent triads available, however, it is clear that assumptions about $P(Me|M, F, C)$ may interfere with inference on $P(D|M, F, C, Me)$ when observing $P(M, F, C, Me|D)$ only. Below, we present the details on the most important situations where this becomes relevant.

We start by briefly mentioning standard approaches to estimating G×Me interactions without using parental data.

Case and control children If only case and control children are available, a standard approach would be to look for interactions using a logistic regression. We have

$$P(C, Me|D) = \frac{P(D|C, Me)P(Me|C)P(C)}{P(D)} \tag{S.4}$$

and

$$P(C, Me|\bar{D}) = \frac{P(\bar{D}|C, Me)P(Me|C)P(C)}{P(\bar{D})}; \tag{S.5}$$

by standard results for log-linear models[1], the odds ratios from a logistic regression of $P(D|C, Me)$ will correspond to the results obtained from the log-linear model, conditioning on D and $\bar{D}$. When assuming rare disease, these odds ratios will be approximately equal to the relative risks in the penetrance model (S.1). This can also be seen from the ratio

$$\frac{P(C, Me|D)}{P(C, Me|\bar{D})} = \frac{P(D|C, Me)}{P(\bar{D}|C, Me)} \cdot \frac{P(\bar{D})}{P(D)} \approx P(D|C, Me) \cdot \frac{1}{P(D)},$$

where $P(D)$ is again treated as a constant in the regression.

Case children only Equation (S.4) contains the term $P(Me|C)$, which depends on combinations of Me and C. If only case children are available, it cannot be separated from $P(D|C, Me)$. That is, if a dependency is found between methylation and child genotype among case children, it cannot be established whether there is an interaction, or whether it simply reflects dependencies in the background population. However, under the additional assumption that

$$P(Me|C) = P(Me), \tag{S.6}$$

i.e., that genotype and methylation are independent in the background population, interactions can be estimated using relation (S.4) alone. While relative risks themselves, such as $RR_2^{(m)} = P(D|C = A_1A_2, Me = m)/P(D|C = A_1A_1, Me = m)$, cannot be estimated without access to controls, the ratios of relative risks, such as $RR_2^{(2)}/RR_2^{(1)}$, can be estimated. This interaction approach is the well-known case only-design[2].

Case-parent triads Even without independent controls, case-parent triad data allow interactions to be assessed and it is achieved with less restrictive assumptions than the case-only design. The case-parent triad relationship (S.2) extends the case-children-only situation (S.4) to include parents. In the standard log-linear model, it is typically assumed that the penetrance does not depend on parental genotypes, i.e., $P(D|M, F, C) = P(D|C)$[3,4]. While this will not be the case if, for instance, maternal genes are involved in disease risk, it is often a tenable assumption. Since Me measures methylation in the child, not the parents, it seems reasonable to extend this to the condition

$$P(D|M, F, C, Me) = P(D|C, Me), \tag{S.7}$$

thus ignoring parental genotypes. As with (S.6), an assumption that $P(Me|M, F, C) = P(Me)$, i.e., that methylation is independent of triad genotypes, would simplify the case-parent triad formula (S.2). However, with parents available, we can make a much less stringent assumption. In fact, it is sufficient to assume that

$$P(Me|M, F, C) = P(Me|M, F), \tag{S.8}$$

i.e., that the methylation in the child may be associated with (parental) genotypes in the population, but that the child's genotype does not directly influence methylation, conditional on parental genotypes. Under the conditions in Equations (S.7) and (S.8), Equation (S.2) becomes

$$\begin{aligned} P(M, F, C, Me|D) &= \frac{P(D|C, Me)P(Me|M, F)P(M, F, C)}{P(D)} \\ &= \frac{P(D|C, Me)P(M, F|Me)P(Me)P(C|M, F)}{P(D)}. \end{aligned}$$

In that case, the term $P(D|C, Me)P(M, F|Me)P(C|M, F)$ corresponds to a stratum-specific log-linear model. $P(Me)$ and $P(D)$ are constant within each stratum and are thus irrelevant to the stratum-specific model.

While the case only-design needs the assumption of independence between genes and methylation, and is thus vulnerable to population stratification, conditioning on parental genotypes prevents a population association between genes and methylation from distorting the interaction analyses. This is a reasonable assumption made in standard G×E analyses based on case-parent triads[5]. It should also be noted that the parental genotypes might have a different within-stratum distribution $P(M, F|Me)$ than $P(M, F)$ in the population at large, precisely if there is population stratification that causes a population association between genotypes and methylation. It might also be caused by, for instance, maternal genes M that increase the mother's proneness to smoking, which again influences her child's methylation patterns. But again, this would not necessarily break condition (S.8), since maternal smoking habits may only be related to her own genes, and not to which allele is passed on to her child.

However, in our situation, where the "environmental exposure" is methylation (Me), one needs to be cautious because it is conceivable that this condition does not hold in situations where the methylation levels at a CpG are directly influenced by a neighboring SNP, as is the case for meQTLs.

Using the full hybrid design of case-parent and control-parent triads In situations where control-parent triads are available in addition to case-parent triads, and under the rare disease assumption, we can use relation (S.3) to estimate $P(Me|M, F, C)$ directly among controls, thus obviating the need for condition (S.8) altogether. Control triads may thus be used just to check if there is an interaction pattern among controls, which would mean that it is most likely due to a meQTL. Alternatively, the estimate obtained from relation (S.2) could be adjusted to account for patterns among controls, by including both case and control triads in a hybrid analysis. In the present setting, we use control-parent triads, when available, as independent checks of plausibility.

References

[1] Agresti, A. (2002). Categorical Data Analysis. (New Jersey: John Wiley & Sons).

[2] Piegorsch, W. W., Weinberg, C. R., and Taylor, J. A. (1994). Non-hierarchical logistic models and case-only designs for assessing susceptibility in population-based case-control studies. Statistics in Medicine *13*, 153–162.

[3] Weinberg, C. R., Wilcox, A. J., and Lie, R. T. (1998). A log-linear approach to case-parent-triad data: assessing effects of disease genes that act either directly or through maternal effects and that may be subject to parental imprinting. The American Journal of Human Genetics *62*, 969–978.

[4] Gjessing, H. K. and Lie, R. T. (2006). Case-parent triads: Estimating single- and double-dose effects of fetal and maternal disease gene haplotypes. Annals of Human Genetics *70*, 382–396.

[5] Umbach, D. M. and Weinberg, C. R. (2000). The use of case-parent triads to study joint effects of genotype and exposure. American Journal of Human Genetics *66*, 251–261.
